# Multivariate Genome-Wide Association Analysis Identifies Genetic Susceptibility Loci for Metabolic Syndrome in the Korean Genome and Epidemiology Study

**DOI:** 10.3390/ijms27188041

**Published:** 2026-09-09

**Authors:** Dasom Kim, Junho Cha, Sungkyoung Choi

**Affiliations:** 1 Department of Mathematical Data Science, College of Computing, Hanyang University, 55 Hanyang-Daehak-ro, Sangnok-gu, Ansan 15588, Republic of Korea; ektha20@hanyang.ac.kr; 2 Department of Applied Artificial Intelligence, College of Computing, Hanyang University, 55 Hanyang-Daehak-ro, Sangnok-gu, Ansan 15588, Republic of Korea; chajunho822@hanyang.ac.kr

**Keywords:** metabolic syndrome, genome-wide association study, multivariate analysis, Korean Genome and Epidemiology Study (KoGES), MPAT, GEMMA

## Abstract

Metabolic syndrome (MetS), characterized by a cluster of interrelated metabolic abnormalities including central obesity, elevated blood pressure, dysglycemia, hypertriglyceridemia, and reduced high-density lipoprotein cholesterol (HDL-C), substantially increases type 2 diabetes and cardiovascular disease risk. Conventional genome-wide association studies (GWASs) analyze individual traits or a dichotomized MetS status, only partially capturing its heritability and potentially overlooking variants with shared (pleiotropic) effects across correlated traits. Here, we performed a multiple-trait GWAS using data from the Korean Genome and Epidemiology Study. Using the Korea Biobank Array (K-Chip), we analyzed a discovery cohort from the Ansan and Ansung studies (1490 cases and 3856 controls) and validated the findings in CAVAS (3620 cases and 4461 controls) and HEXA (15,257 cases and 41,807 controls) cohorts. We jointly modeled six MetS-related traits using the complementary multivariate frameworks Multiple Phenotype Association Tests and Genome-wide Efficient Mixed Model Association, alongside a baseline logistic regression. Whereas logistic regression detected 2 *APOA5* variants (rs662799 and rs2075291), the multivariate analyses identified 79 significant single-nucleotide polymorphisms; all were replicated in the HEXA cohort. Functional annotation prioritized 27 high-risk variants mapping to 12 genes, whereas pathway analysis (DAVID) implicated eight Gene Ontology and Kyoto Encyclopedia of Genes and Genomes pathways related to lipid metabolism. Our findings demonstrate that multivariate analysis substantially improves the identification of pleiotropic susceptibility loci for MetS in the Korean population and indicates candidate genes for early detection and management.

## 1. Introduction

Metabolic syndrome (MetS) comprises a combination of interrelated risk factors including central obesity, elevated fasting glucose, high blood pressure, and dyslipidemia, which collectively increase the risk of type 2 diabetes (T2D) and cardiovascular diseases such as coronary artery disease and cerebrovascular events [1]. Since insulin resistance, driven by both environmental factors (obesity, diet, and physical inactivity) and genetic predisposition, is regarded as the central pathophysiological mechanism, its multifactorial complexity has made characterizing the genetic architecture of MetS difficult.

The MetS burden has risen steadily worldwide, reflecting Westernized dietary patterns, population aging, and increasingly sedentary lifestyles. In Korea, national surveillance has reported that the age-adjusted prevalence has increased from 24.9% in 1998 to 31.3% in 2007 [2]. More recent surveys estimate a 20–30% prevalence among Korean adults, with a continued rise among male individuals [3,4]. Comparable trends are evident across the Asia-Pacific region, where nearly one-fifth of adults are affected [5]. Twin studies have estimated the heritability of MetS at 0.51–0.60, indicating a substantial genetic contribution and the clinical importance of identifying susceptibility variants [6].

Genome-wide association studies (GWASs) have identified numerous loci for MetS and its individual components. In Korean cohorts, variants in genes such as *APOA5*, *ZPR1*, *BUD13*, *GCKR*, *ALDH1A2*, and *LIPC* have been linked to metabolic traits [7], and pathway analyses have implicated lipid metabolism and related processes [8]. Nevertheless, the variants identified to date only partially explain MetS heritability—the so-called missing heritability—partly because conventional analyses focus on common variants of small effect and rarely capture the interactions among the factors influencing MetS risk [9].

Most previous genetic studies on MetS adopted a univariate framework, analyzing each component separately or as a binary diagnosis. However, this approach has several important limitations. First, dichotomizing continuous measurements discards information and reduces statistical power. Second, single-trait analyses ignore the correlations among metabolic traits that may share genetic determinants. Third, pleiotropic variants exerting moderate effects across several correlated traits may be missed when each trait is tested in isolation. Joint analysis of multiple correlated traits can overcome these constraints and enhance the power to detect associations [10].

Linear mixed models have become the standard framework for GWASs because they control for population stratification and cryptic relatedness [11], and their multivariate extension simultaneously analyzes multiple traits while accounting for genetic relatedness and phenotypic correlation [12]. A complementary class of methods instead combines univariate (per-trait) summary statistics while modeling the between-trait correlation, which accommodates diverse genetic architectures and effect direction [13,14]. Therefore, we leveraged two multivariate approaches in this study: Multiple Phenotype Association Test (MPAT) [14] and the Genome-wide Efficient Mixed Model Association (GEMMA), which fits an individual-level multivariate linear mixed model [11,12].

Allele frequencies, linkage disequilibrium patterns, and gene–environment interactions differ across ancestries; therefore, findings from European-ancestry studies may not generalize to East Asian populations [15,16]. Motivated by this need for population-specific analyses, the aim of this study was to identify genetic susceptibility loci for metabolic syndrome in a Korean population. Specifically, we sought (i) to detect susceptibility variants by jointly analyzing the correlated traits that define MetS rather than a single dichotomized outcome; (ii) to validate the identified variants in two independent replication cohorts; and (iii) to characterize the prioritized variants through functional annotation and pathway analysis to gain mechanistic insight into MetS pathogenesis.

## 2. Results

### 2.1. Characteristics of the Study Population

We analyzed three independent KoGES cohorts genotyped with the Korea Biobank Array (Table 1). After quality control, the discovery cohort (Ansan and Ansung) comprised 1490 MetS cases and 3856 controls, replication cohort I (CAVAS) comprised 3620 cases and 4461 controls, and replication cohort II (HEXA) comprised 15,257 cases and 41,807 controls. Across all three cohorts, cases were significantly older than controls and differed significantly in sex distribution (*p* < 0.001). MetS status was defined according to the NCEP ATP III criteria [17]. Demographic characteristics of the three cohorts are summarized in Table 1.

The three cohorts were recruited using different designs, comprising the community-based Ansan and Ansung and CAVAS studies and the urban health-examinee HEXA study, and they differed in age and sex structure. Therefore, they were analyzed as independent discovery and replication sets rather than pooled, and a formal statistical comparison between the cohorts was beyond the scope of this replication-based design.

### 2.2. Baseline Logistic Regression Analysis

**Figure 1:**
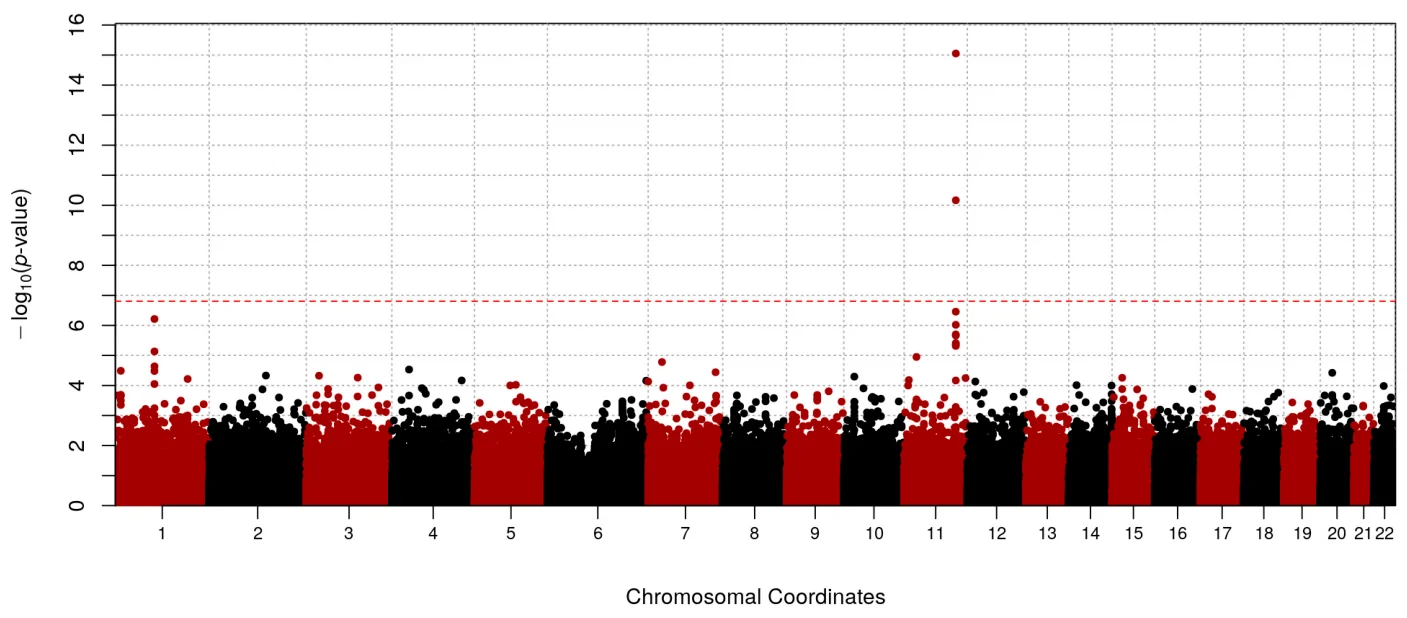
Manhattan plot of the logistic regression analysis for metabolic syndrome (MetS) in the discovery cohort. The *x*-axis shows the chromosomal position, and the *y*-axis presents the −log_10_(*P*); the horizontal line denotes the genome-wide significance threshold (Bonferroni correction, *p* < 1.564 × 10^−7^). Alternating colors distinguish adjacent chromosomes.

Using logistic regression with binary MetS status in the discovery cohort, we identified two genome-wide significant single-nucleotide polymorphisms (SNPs) (Bonferroni-corrected *p* < 1.564 × 10^−7^), rs662799 and rs2075291 (Figure 1), which are located within *APOA5*. The quantile–quantile plots for each cohort showed no evidence of genomic inflation (Figure S1).

In addition to the two genome-wide significant *APOA5* variants, a suggestive signal on chromosome 1 approached but did not reach the significance threshold (*p* < 1.564 × 10^−7^); the lead variant at this locus, rs4554698 (chr1:88513504, nearest gene *RPL36AP10*), is reported here as a suggestive association.

### 2.3. Multivariate Association Analysis Using MPAT and GEMMA

We next jointly analyzed the six MetS-related traits. Using the MPAT approach, we identified 34 significant SNPs in the discovery cohort (Bonferroni-corrected *p* < 1.564 × 10^−7^): 4 using the SUM method, 9 using the VC method, and 34 using the mix-Fisher method, which encompassed the SUM and VC results. The two *APOA5* variants (rs662799 and rs2075291) were consistently detected using all three MPAT methods. The quantile–quantile plots of the three MPAT statistics closely followed the null expectation in the discovery and two replication cohorts; deviations were confined to the extreme tail, indicating well-calibrated tests with negligible genomic inflation (Figure S2). All 34 MPAT SNPs were replicated in the HEXA cohort, and 23 were also replicated in the CAVAS cohort (Table S1). The Bonferroni-corrected thresholds for the two replication cohorts were 1.564 × 10^−7^ and 1.566 × 10^−7^, respectively.

**Figure 2:**
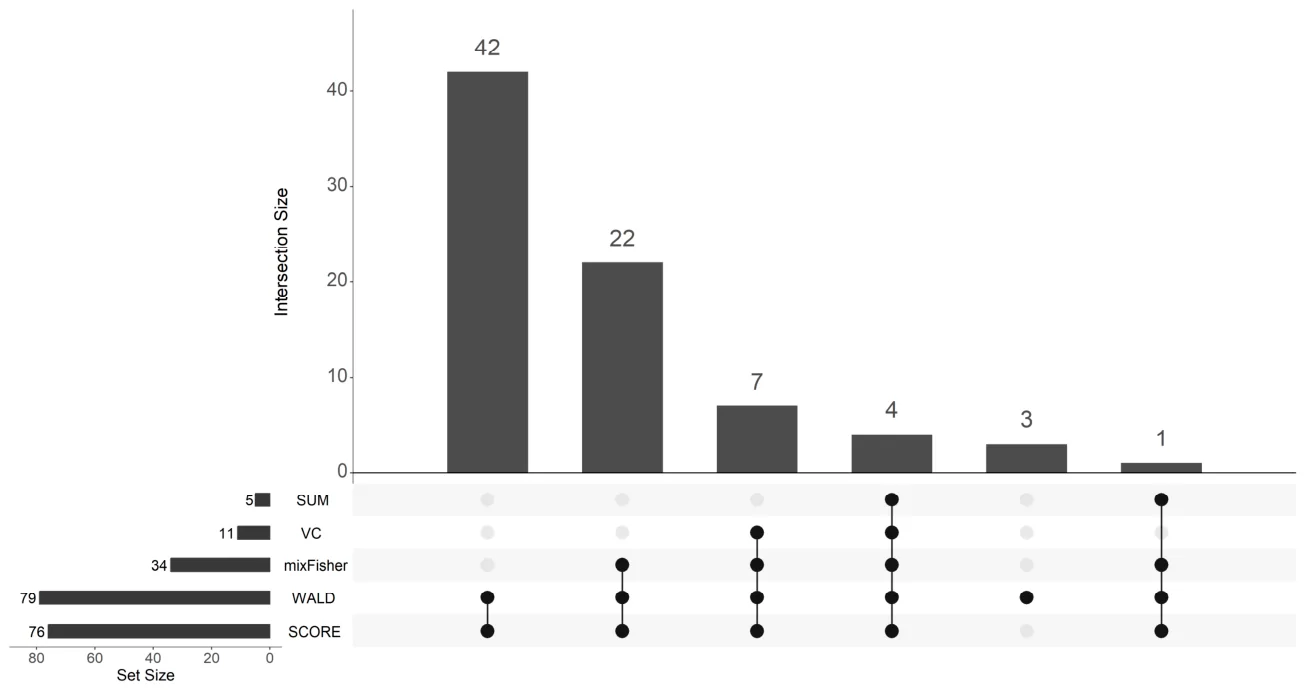
UpSet plot showing the overlap of significant single-nucleotide polymorphisms (SNPs) across the Multiple Phenotype Association Test (MPAT) (SUM, VC, and mix-Fisher) and Genome-wide Efficient Mixed Model Association (GEMMA) (WALD and SCORE) methods. Vertical bars indicate the number of SNPs in each intersection, and connected dots denote the methods contributing to that intersection.

**Figure 3:**
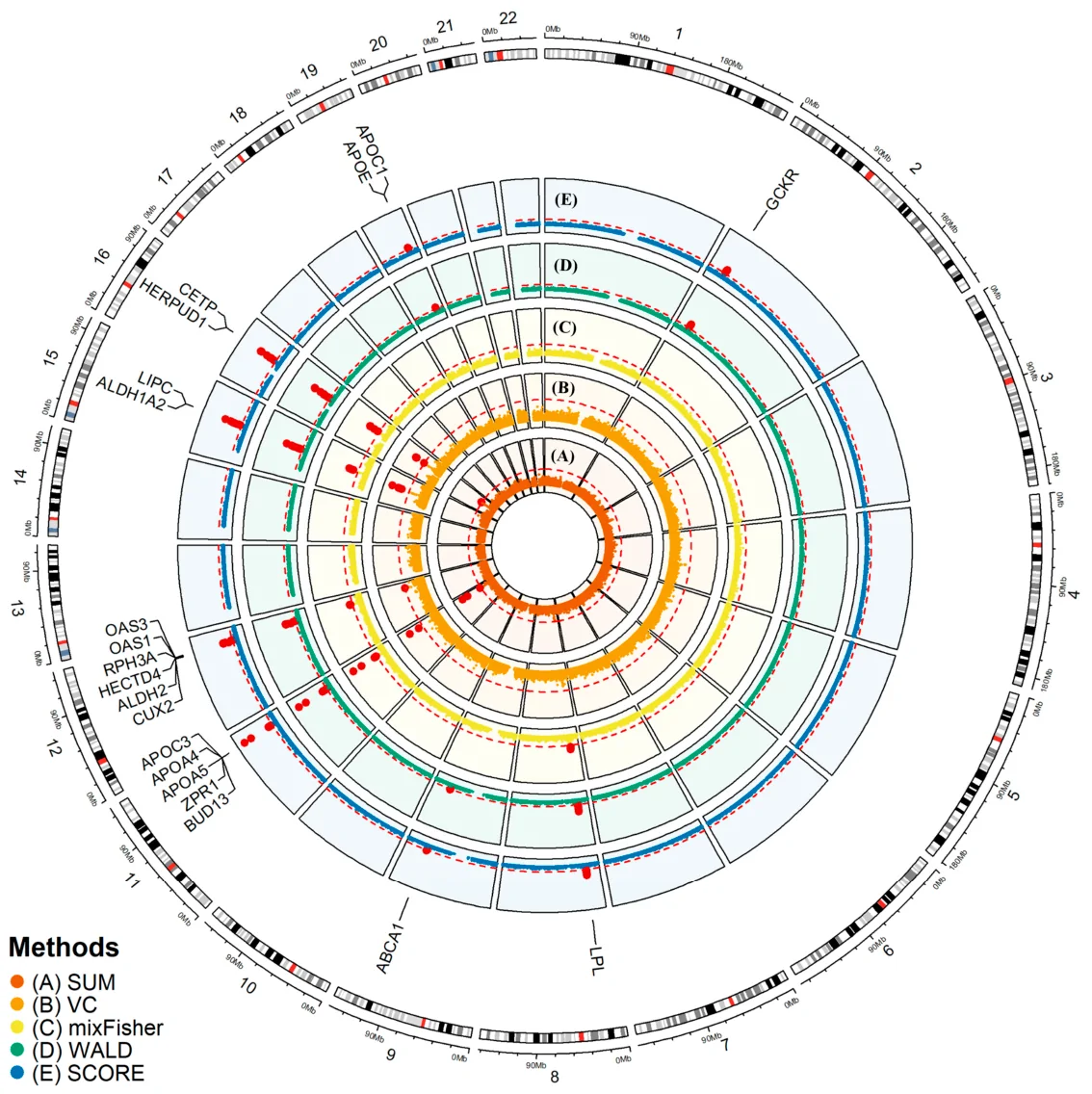
Circular Manhattan plot showing SNP distribution and their corresponding chromosomal locations across the five multivariate analyses in the discovery cohort: (**A**) SUM of MPAT, (**B**) VC of MPAT, (**C**) mix-Fisher of MPAT, (**D**) WALD of GEMMA, and (**E**) SCORE of GEMMA. Each point represents the −log_10_(*P*). The red dashed line indicates the significance threshold (Bonferroni correction, *p* < 1.564 × 10^−7^). Red dots denote the 79 significant SNPs associated with MetS, and the genes mapped to these SNPs are labeled outside the plot.

For the GEMMA method, the likelihood ratio test showed an inflationary pattern in the discovery cohort (Figure S3) and was thus excluded from downstream analysis. The WALD and SCORE tests were retained and identified 79 and 76 significant SNPs, respectively (Bonferroni-corrected *p* < 1.564 × 10^−7^). All 79 GEMMA variants were replicated in the HEXA cohort, whereas 64 were replicated in the CAVAS cohort; moreover, the GEMMA score test encompassed all 34 SNPs identified using MPAT. Four SNPs were significant across all five multivariate analyses (Figure 2). In total, the multivariate analyses identified 79 significant SNPs associated with MetS. The chromosomal distribution across the five methods is shown in the integrated circular Manhattan plot (Figure 3).

No chromosome-1 variant reached the genome-wide significance threshold in the multivariate analyses; therefore, there is no highlighted signal for chromosome 1 in Figure 3. This contrasts with the near-threshold chromosome-1 signal (rs4554698) observed in the univariate logistic analysis (Figure 1). Such a difference is expected because the multivariate tests are designed to capture variants with shared (pleiotropic) effects across the six correlated traits, whereas the chromosome-1 association appears to be driven mainly by the binary MetS classification rather than by a coordinated effect across the individual traits.

### 2.4. Functional Annotation and Prioritization of Risk Variants

We annotated the 79 significant variants with ANNOVAR (version 2018Apr16, https://annovar.openbioinformatics.org/en/latest/; accessed on 7 July 2026) with the hg19/GRCh37 reference genome and Ensemble gene build 106 [18] and assessed their potential pathogenicity with CADD [19], DANN [20], and RegulomeDB [21]. The RegulomeDB classification system is summarized in Table S2. Applying the high-risk thresholds (CADD ≥ 12.37, DANN > 0.8, or RegulomeDB rank ≤ 3) prioritized 27 high-risk SNPs (Table 2), 21 of which had been previously reported as MetS-associated. Ten of the prioritized variants were expression quantitative trait loci (eQTLs), and the genes mapped to these eQTLs (*GCKR*, *LPL*, *APOC3*, and *ZPR1*) are established MetS risk genes [22,23,24,25,26,27,28].

Among the 27 prioritized variants, six were not previously reported in association with MetS or its components and thus represent novel candidates: rs13702, rs271, rs28526159, and rs7841189 (*LPL*); rs11066359 (*RPH3A*); and rs28528324 (*ALDH1A2*). Notably, four of the six localize to *LPL*, a central regulator of triglyceride lipolysis, and the two most deleterious by CADD—rs13702 (*LPL* 3′-UTR; CADD = 43.0) and rs11066359 (*RPH3A*; CADD = 35.0)—are strong candidates for independent replication and functional validation.

### 2.5. Pathway Analysis

Pathway analysis with DAVID was performed on the 12 genes (*GCKR*, *LPL*, *APOC3*, *LIPC*, *CETP*, *ZPR1*, *HECTD4*, *OAS3*, *BUD13*, *ALDH1A2*, *HERPUD1*, and *RPH3A*) mapped to the 27 high-risk SNPs. This analysis yielded eight significant Gene Ontology (GO) terms and Kyoto Encyclopedia of Genes and Genomes (KEGG) pathways (false discovery rate correction *q*-value < 0.05), all related to triglyceride, low-density lipoprotein (LDL), high-density lipoprotein, and cholesterol metabolism (Table 3). Genes including *GCKR* [25,28,108], *LPL* [24,26,27,109,110,111], *APOC3* [22,23,64], *LIPC* [93,112,113], and *CETP* [105,114,115,116,117] recurred across these pathways, consistent with their established roles in MetS, T2D, obesity, and cardiovascular disease.

## 3. Discussion

In this study, we identified genetic susceptibility loci for MetS in a Korean population by jointly analyzing six correlated MetS-related traits with two complementary multivariate frameworks. While baseline logistic regression using binary MetS status detected two *APOA5* variants (rs662799 and rs2075291) that are both previously reported MetS risk factors that have been repeatedly associated with MetS [7,140,141,142,143], the multivariate analyses substantially expanded discovery [144,145,146], identifying 34 and 79 SNPs using MPAT and GEMMA, respectively. This marked increase illustrates the power gained by modeling the shared genetic architecture of correlated metabolic traits rather than a single dichotomized outcome.

The MPAT and GEMMA approaches are methodologically complementary. MPAT estimates the phenotypic correlation matrix and combines univariate summary statistics to detect both homogeneous (SUM) and heterogeneous (VC) effects, with the mix-Fisher test jointly capturing both. Meanwhile, GEMMA fits a multivariate linear mixed model that accounts for random effects and cryptic relatedness, enabling exact computation of the test statistics. Our findings that all 34 MPAT SNPs were recovered within the GEMMA score test results and 4 SNPs were significant across all five analyses support the robustness of the identified associations. The consistent replication of variants in the large HEXA cohort further strengthens confidence in these findings.

Functional annotation prioritized 27 high-risk variants mapping to 12 genes, and pathway analysis implicated eight pathways governing triglyceride, LDL, HDL-C, and cholesterol metabolism. The recurrent genes—*GCKR*, *LPL*, *APOC3*, *LIPC*, and *CETP*—are well-established regulators of lipid metabolism that have been repeatedly linked to MetS, T2D, obesity, and cardiovascular disease [7,23,25,29,30,31,32,33,34,35,36,37,38,39,40,41,42,43,44,45,46,47,48,49,50,51,52,53,54,55,56,57,58,59,61,62,63,64,65,66,67,68,69,70,89,90,91,102,103,104,105,106,107]. eQTL enrichment among the prioritized variants suggests that regulatory effects on gene expression may contribute to MetS susceptibility, offering potential mechanistic insight.

The convergence of the implicated genes on lipid metabolism—despite MetS extending to the glycemic, blood-pressure, and adiposity domains—likely reflects several factors: the two lipid traits (TG and HDL-C) carry relatively high heritability and larger per-variant effects among the six components, and the pleiotropy-oriented multivariate tests preferentially capture variants with shared effects. For MetS, these are enriched at lipid loci, while the glycemic, blood-pressure, and central-obesity components are more polygenic with smaller per-variant effects, so their loci are less likely to pass the stringent multivariate threshold. Trait-stratified and larger multi-ancestry analyses will be needed to resolve non-lipid contributions. These findings are concordant with previous Korean and East-Asian MetS GWASs—for example, associations at *APOA5/ZPR1/BUD13*, *LPL*, *GCKR*, *CETP*, and *LIPC* were reported by Lee et al. [1], Oh et al. [7], and Shim et al. [8]. Importantly, whereas the univariate logistic analysis detected only the *APOA5* signal, the multivariate framework additionally resolved multiple correlated-trait (pleiotropic) variants at these loci, underscoring the added value of jointly modeling correlated traits.

By nominating a panel of candidate risk variants through multivariate analysis, our approach may improve the classification and prediction of MetS risk and support its early detection and management. Moreover, the analytical strategy is broadly applicable: multivariate methods such as MPAT and GEMMA can enhance the identification of genetic susceptibility for other complex diseases characterized by correlated phenotypes.

This study has some limitations. As the analysis was restricted to a Korean population, certain variants may have been missed. Caution is warranted when generalizing the findings to populations of other ancestries, and larger and more diverse samples are needed. Additionally, we focused on common variants, but rare variants may account for a substantial portion of the unexplained heritability of MetS, and their omission may limit predictive accuracy. Future work incorporating rare-variant and cross-ancestry analyses will help complete the genetic picture of MetS.

Finally, because MetS is defined by the presence of at least three of five diagnostic criteria, there are 16 theoretically distinct trait combinations (ten with three of five, five with four of five, and one with all five); subtype- or combination-specific multivariate analyses therefore represent a promising direction for dissecting the heterogeneous genetic architecture of MetS.

## 4. Materials and Methods

### 4.1. Study Participants

This study analyzed de-identified secondary data from the Korean Genome and Epidemiology Study (KoGES), a national cohort established by the Korea Disease Control and Prevention Agency (KDCA) through the Korean Genome Analysis Project consortium. All KoGES participants provided written informed consent to the KoGES investigators at the time of enrollment, including consent for the secondary use of their de-identified data in approved research. The datasets were provided to our team through the official KDCA data-distribution procedure following review of our IRB-approved research plan, and all analyses were conducted within the KDCA’s secure Clinical and Omics Data Archive (CODA) system. Ethical considerations of this study were reviewed and approved by the Institutional Review Board of Hanyang University (IRB No. 275, HYUIRB-202606-004), which waived the requirement for additional informed consent. The study was conducted in accordance with the Declaration of Helsinki.

### 4.2. Phenotype and Trait Definition

MetS was defined according to the final report of the NCEP ATP III [17] as the presence of at least three of the following five components: (i) WC ≥ 90 or ≥80 cm for male or female individuals, respectively; (ii) TG ≥ 150 mg/dL or treatment for dyslipidemia; (iii) HDL-C < 40 or <50 mg/dL for male or female individuals, respectively, or treatment for dyslipidemia; (iv) SBP ≥ 130 mmHg, DBP ≥ 85 mmHg, or treatment for hypertension; and (v) FPG ≥ 100 mg/dL or treatment for diabetes mellitus.

To compare analytical strategies, we adopted two complementary representations of MetS. For baseline comparisons, MetS was modeled as a binary outcome (case vs. control) and tested using logistic regression. For the main multiple-trait analysis, we jointly modeled six continuous MetS-related traits: WC, SBP, DBP, log(TG), log(FPG), and −log(HDL-C). This approach enabled us to preserve the quantitative information and correlation structure among traits that a binary definition would otherwise discard. TG, FPG, and HDL-C were log- or negative-log-transformed to reduce skewness. All analyses were adjusted for age (a continuous variable), sex (male or female), and area (only for the discovery cohort: Ansan or Ansung) as covariates.

### 4.3. Genotyping and Quality Control

DNA samples were genotyped using the Korea Biobank Array (Korean Chip, KORV1.1) designed by the Korea National Institute of Health, Cheongju, Republic of Korea; genotyped on the Axiom platform (Thermo Fisher Scientific, Santa Clara, CA, USA) [147]. Quality control was performed using PLINK v1.9.0 software (National Institutes of Health, Bethesda, MD, USA) [148], applying the following exclusion thresholds: genotype missing call rate > 0.05, individual missing rate > 0.05, minor allele frequency < 0.05, and Hardy–Weinberg equilibrium test *p* ≤ 1 × 10^−6^. After quality control filtering, a comparable number of SNPs was retained across the three cohorts: 319,640 (discovery cohort), 319,706 (CAVAS cohort), and 319,317 (HEXA cohort).

### 4.4. Statistical Analyses

We tested associations between genetic variants and MetS using three approaches: a baseline logistic regression on binary MetS status and two multiple-trait methods (MPAT and GEMMA) that jointly analyzed the six MetS-related traits.

#### 4.4.1. Logistic Regression

Logistic regression was fitted in PLINK v1.9.0 software [148], modeling binary MetS status as a function of the covariates and each SNP, with a Bonferroni-corrected significance threshold of *p* < 1.564 × 10^−7^.

#### 4.4.2. MPAT

To account for phenotypic correlations, MPAT was applied using three tests: SUM, which identifies homogeneous effects across traits using summary statistics; VC, which is more robust than SUM when the effects are heterogeneous; and mix-Fisher, which jointly tests homogeneous and heterogeneous genetic effects [14].

Specifically, MPAT estimates that phenotypic correlation matrix from the residualized traits and combines the per-trait univariate summary statistics accordingly: the SUM statistic aggregates signed univariate statistic (most powerful under concordant, homogeneous effects), the VC statistic is a variance-component test that remains robust under heterogeneous effects, and the mix-Fisher statistic combines the SUM and VC *p*-values by Fisher’s method to capture both. The precise test statistics and their null distributions are defined in the Supplementary Methods (Text S1).

#### 4.4.3. GEMMA

GEMMA was used to fit a multivariate linear mixed model relating the variants to the multiple traits while accounting for a genetic relatedness matrix and environmental covariance [11,12]. *p*-values were computed using the WALD, likelihood ratio, and SCORE tests. The likelihood ratio test was excluded from downstream analysis because of genomic inflation in the discovery cohort. The Bonferroni-corrected thresholds were *p* < 1.564 × 10^−7^ for the discovery and CAVAS cohorts and *p* < 1.566 × 10^−7^ for the HEXA cohort.

In this model, the six traits are jointly regressed on genotype and covariates, with a centered genetic relatedness matrix modeling random polygenic effects and a residual covariance matrix modeling the correlation among traits, thereby controlling for population structure and cryptic relatedness. Additional details are given in the Supplementary Methods (Text S1).

#### 4.4.4. Functional Annotation and Pathway Analysis

Significant variants were annotated with ANNOVAR (version 2018Apr16, https://annovar.openbioinformatics.org/en/latest/; accessed on 7 July 2026) with the hg19/GRCh37 reference genome and Ensemble gene build 106 [18] and their potential pathogenicity with CADD [19], DANN [20], and RegulomeDB [21]. High-risk variants were defined as CADD ≥ 12.37, DANN > 0.8, or RegulomeDB rank ≤ 3. GO enrichment and KEGG pathway analyses of genes mapped to the high-risk variants were performed using DAVID [149], with significance defined as a false discovery rate (*q*-value) < 0.05.

The following software were used, with versions and sources as indicated: PLINK v1.9.0 (https://www.cog-genomics.org/plink/); GEMMA version 3 (University of Michigan, Ann Arbor, MI, USA); ANNOVAR version 2018Apr16 (Wang Genomics Lab, University of Southern California, Los Angeles, CA, USA); CADD version 1.3 (University of Washington, Seattle, WA, USA); DANN version 1.0 (University of California, Irvine, CA, USA); RegulomeDB version 2.2 (Stanford University, Stanford, CA, USA); and DAVID version 6.8 (Laboratory of Human Retrovirology and Immunoinformatics, Frederick, MD, USA).

## 5. Conclusions

Using a multiple-trait GWAS of the KoGES, we jointly modeled six correlated MetS-related traits with MPAT and GEMMA and identified 79 genetic variants associated with MetS that were undetected by conventional logistic regression. Functional annotation and pathway analysis prioritized 27 high-risk variants and 12 genes that converge on lipid- and cholesterol-metabolism pathways. These findings highlight the value of multivariate analysis for uncovering pleiotropic susceptibility loci and provide candidate genetic markers to inform the early detection and management of MetS.

## Figures and Tables

**Table 1:** Demographic and clinical characteristics of the study participants across the discovery and two replication cohorts.

Characteristics	Discovery Cohort (Ansan and Ansung)			Replication Cohort I (CAVAS)			Replication Cohort II (HEXA)		
	Case (*n* = 1490)	Control (*n* = 3856)	*p*-Value	Case (*n* = 3620)	Control (*n* = 4461)	*p*-Value	Case (*n* = 15,257)	Control (*n* = 41,807)	*p*-Value
AGE (years) ^a^	54.1 ± 8.2	50.3 ± 8.3	<0.001	59.5 ± 8.2	57.5 ± 9.2	<0.001	56.6 ± 7.6	52.8 ± 7.9	<0.001
Sex ^b^			<0.001			<0.001			<0.001
Male	546 (36.6%)	2006 (52.0%)		1223 (33.8%)	1825 (40.9%)		5834 (38.2%)	13,861 (33.2%)	
Area			<0.001						
Ansan	881 (59.1%)	1642 (42.6%)							
Ansung	609 (40.9%)	2214 (57.4%)							
WC ^c^	88.9 ± 7.0	79.9 ± 7.8	<0.001	88.5 ± 7.5	80.9 ± 8.1	<0.001	86.8 ± 7.6	78.6 ± 7.9	<0.001
TG ^d^	223.6 ± 118.8	135.7 ± 84.8	<0.001	191.3 ± 112.8	109.9 ± 54.4	<0.001	185.7 ± 113.7	103.0 ± 58.5	<0.001
HDL-C ^e^	39.2 ± 6.9	46.9 ± 10.0	<0.001	40.8 ± 8.9	48.6 ± 11.1	<0.001	46.1 ± 10.8	56.6 ± 12.8	<0.001
SBP ^f^	127.3 ± 18.6	112.4 ± 15.4	<0.001	130.8 ± 16.9	119.5 ± 16.1	<0.001	130.4 ± 14.2	119.5 ± 13.9	<0.001
DBP ^g^	81.0 ± 11.7	72.5 ± 10.3	<0.001	81.8 ± 10.7	76.0 ± 10.2	<0.001	79.9 ± 9.4	74.2 ± 9.4	<0.001
FPG ^h^	94.4 ± 27.8	84.5 ± 15.2	<0.001	104.8 ± 24.8	92.6 ± 14.3	<0.001	105.8 ± 27.1	91.2 ± 14.2	<0.001

^a^ Means  ±  standard deviation (SD); ^b^ number (%); ^c^ waist circumference (cm); ^d^ triglyceride (mg/dL); ^e^ high-density lipoprotein cholesterol (mg/dL); ^f^ systolic blood pressure (mmHg); ^g^ diastolic blood pressure (mmHg); ^h^ fasting glucose levels (mg/dL).

**Table 2:** Functional annotation of the 27 high-risk SNPs.

Chr ^a^	rsID ^b^	Ref ^c^	Alt ^d^	Gene Function	Nearest Genes	CADD ^e^	DANN ^f^	RDB Rank ^g^	References
2	rs780092	A	G	intron	*GCKR*	1.816	0.468	1d	[29,30]
2	rs780093	T	C	intron	*GCKR*	7.898	0.847	4	[25,31,32,33,34]
2	rs780094	T	C	intron	*GCKR*	3.086	0.608	2c	[25,35,36,37,38,39,40,41]
8	rs10503669	C	A	downstream	*LPL*	14.720	0.776	1f	[42,43,44]
8	rs13702	T	C	UTR-3	*LPL*	43.000	0.996	5	-
8	rs17411031	C	G	downstream	*LPL*	2.863	0.298	1f	[45,46,47]
8	rs17482753	G	T	downstream	*LPL*	0.457	0.670	1f	[47,48,49,50]
8	rs271	G	A	intron	*LPL*	1.380	0.295	1f	-
8	rs28526159	C	T	downstream	*LPL*	6.396	0.824	5	-
8	rs326	A	G	intron	*LPL*	1.905	0.169	1f	[51,52,53,54,55,56,57]
8	rs7016880	G	C	downstream	*LPL*	2.756	0.405	1f	[58,59]
8	rs7841189	C	T	downstream	*LPL*	1.827	0.501	2c	-
11	rs11216126	A	C	downstream	*BUD13*	10.270	0.844	7	[7,60]
11	rs5128	G	G	UTR-3	*APOC3*	4.347	0.407	1b	[23,61,62,63,64,65,66,67,68,69,70]
11	rs603446	C	T	intron	*ZPR1*	-	-	1f	[7,37,60,71]
11	rs6589566	G	G	intron	*ZPR1*	24.500	0.996	6	[71,72,73,74,75,76,77]
11	rs964184	G	G	UTR-3	*ZPR1*	6.308	-	1d	[7,78,79,80,81]
12	rs11066359	C	T	intron	*RPH3A*	35.000	0.999	5	-
12	rs2072134	G	A	UTR-3	*OAS3*	8.625	0.530	3a	[82,83,84]
12	rs2074356	G	A	intron	*HECTD4*	2.838	0.764	3a	[8,85,86]
15	rs10468017	C	T	intron	*ALDH1A2*	0.570	0.659	3a	[7,87,88]
15	rs11635491	G	A	intron	*LIPC*	9.579	0.835	5	[89,90,91]
15	rs1532085	A	G	intron	*ALDH1A2*	1.712	0.811	5	[92,93,94,95,96,97,98]
15	rs2043085	T	C	intron	*ALDH1A2*	11.850	0.847	6	[34,92,93]
15	rs28528324	T	C	intron	*ALDH1A2*	2.454	0.656	3a	-
16	rs247617	C	A	downstream	*HERPUD1*	9.323	0.833	4	[7,99,100,101]
16	rs708272	G	A	intron	*CETP*	0.455	0.388	2b	[7,102,103,104,105,106,107]

^a^ Chromosome; ^b^ hg19, dbSNP150 version; ^c^ Reference allele; ^d^ Alternative allele; ^e^ CADD, Combined Annotation Dependent Depletion (PHRED-scaled) score; higher scores indicate greater predicted deleteriousness (a PHRED score of 10 or 20 corresponds to the top 10% or top 1% most deleterious substitutions genome-wide, respectively), and a CADD PHRED score ≥ 12.37 was applied as the high-risk threshold; ^f^ the DANN score ranges from 0 to 1, where 1 represents the highest possibility for pathogenicity; ^g^ scores range from 1 to 7, with lower values indicating that the variant is more likely to be located in the functional region. Variants marked “-” in the References column were not previously reported in association with MetS or its components and are therefore considered novel: rs13702, rs271, rs28526159, and rs7841189 (*LPL*); rs11066359 (*RPH3A*); and rs28528324 (*ALDH1A2*).

**Table 3:** Results of the Gene Ontology (GO) and Kyoto Encyclopedia of Genes and Genomes (KEGG) pathway enrichment analyses.

Pathway Description ^a^	Genes	Count (%) ^b^	FDR	References
GO pathway				
GO:0070328~Triglyceride homeostasis	*CETP*, *LIPC*, *LPL*, *APOC3*, *GCKR*	5 (41.7)	5.70 × 10^−7^	[118,119,120,121]
GO:0034375~High-density lipoprotein particle remodeling	*APOC3*, *CETP*, *LIPC*, *LPL*	4 (33.3)	7.20 × 10^−6^	[122,123,124]
GO:0034372~Very-low-density lipoprotein particle remodeling	*CETP, LIPC, LPL*	3 (25.0)	4.40 × 10^−4^	[125,126,127]
GO:0042632~Cholesterol homeostasis	*APOC3, CETP, LIPC, LPL*	4 (33.3)	1.10 × 10^−3^	[128,129,130,131]
GO:0043691~Reverse cholesterol transport	*APOC3, CETP, LIPC*	3 (25.0)	1.40 × 10^−3^	[132,133,134,135]
GO:0019433~Triglyceride catabolic process	*APOC3, LIPC, LPL*	3 (25.0)	1.50 × 10^−3^	[118,119,120,121]
GO:0006641~Triglyceride metabolic process	*APOC3, CETP, LPL*	3 (25.0)	4.40 × 10^−3^	[24,118,119,120,121,136]
KEGG pathway				
map04979~Cholesterol metabolism	*APOC3*, *CETP*, *LIPC*, *LPL*	4 (33.3)	5.50 × 10^−5^	[131,137,138,139]

Results and summary of GO and KEGG pathway analyses using the DAVID tool for 12 genes mapped to 27 SNPs that satisfied the risk-allele criteria among 79 SNPs identified as significant using MPAT and GEMMA. ^a^ Pathway Description provides GO (biological process, BP) and KEGG pathway results, including pathway ID and term information. ^b^ Count (%) indicates the number and percentage of genes identified as significant in our GEMMA analysis results among all genes belonging to each GO term and KEGG pathway.

## Data Availability

This study analyzed the data of KoGES from the Korea Disease Control and Prevention Agency (KDCA). Requests to obtain the datasets should be made to the KDCA (https://coda.nih.go.kr/frt/index.do, accessed on 7 July 2026).

## Supplementary Materials

The following supporting information can be downloaded at: https://www.mdpi.com/article/10.3390/ijms27188041/s1.

## Abbreviations

The following abbreviations are used in this manuscript:

MetS	Metabolic syndrome
T2D	Type 2 diabetes
GWAS	Genome-wide association studies
MPAT	Multiple Phenotype Association Tests
GEMMA	Genome-wide Efficient Mixed Model Association
SD	Standard deviation
SNPs	Single-nucleotide polymorphisms
GO	Gene Ontology
KEGG	Kyoto Encyclopedia of Genes and Genomes
LDL	Low-density lipoprotein
HDL-C	High-density lipoprotein cholesterol
WC	Waist circumference
SBP	Systolic blood pressure
DBP	Diastolic blood pressure
FPG	Fasting plasma glucose

## References

[1] Lee H.S., Kim Y., Park T. (2018). New Common and Rare Variants Influencing Metabolic Syndrome and Its Individual Components in a Korean Population. Sci. Rep..

[2] Lim S., Shin H., Song J.H., Kwak S.H., Kang S.M., Won Yoon J., Choi S.H., Cho S.I., Park K.S., Lee H.K. (2011). Increasing prevalence of metabolic syndrome in Korea: The Korean National Health and Nutrition Examination Survey for 1998–2007. Diabetes Care.

[3] Kim M.-h., Lee S.-h., Shin K.-S., Son D.-Y., Kim S.-H., Joe H., Yoo B.-W., Hong S.-H., Cho C.-Y., Shin H.-S. (2020). The change of metabolic syndrome prevalence and its risk factors in Korean adults for decade: Korea National Health and Nutrition Examination Survey for 2008–2017. Korean J. Fam. Pract..

[4] Huh J.H., Kang D.R., Kim J.Y., Koh K.K. (2021). Metabolic syndrome fact sheet 2021: Executive report. CardioMetab. Syndr. J..

[5] Ranasinghe P., Mathangasinghe Y., Jayawardena R., Hills A.P., Misra A. (2017). Prevalence and trends of metabolic syndrome among adults in the asia-pacific region: A systematic review. BMC Public Health.

[6] Sung J., Lee K., Song Y.M. (2009). Heritabilities of the metabolic syndrome phenotypes and related factors in Korean twins. J. Clin. Endocrinol. Metab..

[7] Oh S.W., Lee J.E., Shin E., Kwon H., Choe E.K., Choi S.Y., Rhee H., Choi S.H. (2020). Genome-wide association study of metabolic syndrome in Korean populations. PLoS ONE.

[8] Shim U., Kim H.N., Sung Y.A., Kim H.L. (2014). Pathway Analysis of Metabolic Syndrome Using a Genome-Wide Association Study of Korea Associated Resource (KARE) Cohorts. Genom. Inform..

[9] Moon S., Lee Y., Won S., Lee J. (2018). Multiple genotype-phenotype association study reveals intronic variant pair on SIDT2 associated with metabolic syndrome in a Korean population. Hum. Genom..

[10] O’Reilly P.F., Hoggart C.J., Pomyen Y., Calboli F.C., Elliott P., Jarvelin M.R., Coin L.J. (2012). MultiPhen: Joint model of multiple phenotypes can increase discovery in GWAS. PLoS ONE.

[11] Zhou X., Stephens M. (2012). Genome-wide efficient mixed-model analysis for association studies. Nat. Genet..

[12] Zhou X., Stephens M. (2014). Efficient multivariate linear mixed model algorithms for genome-wide association studies. Nat. Methods.

[13] Ray D., Pankow J.S., Basu S. (2016). USAT: A Unified Score-Based Association Test for Multiple Phenotype-Genotype Analysis. Genet. Epidemiol..

[14] Liu Z., Lin X. (2018). Multiple phenotype association tests using summary statistics in genome-wide association studies. Biometrics.

[15] Martin A.R., Kanai M., Kamatani Y., Okada Y., Neale B.M., Daly M.J. (2019). Clinical use of current polygenic risk scores may exacerbate health disparities. Nat. Genet..

[16] Jo J., Ha N., Ji Y., Do A., Seo J.H., Oh B., Choi S., Choe E.K., Lee W., Son J.W. (2025). Association of Multiple-trait Polygenic Risk Score with Obesity and Cardiometabolic Diseases in Korean Population. Genom. Proteom. Bioinform..

[17] (2002). National Cholesterol Education Program (NCEP) Expert Panel on Detection, Evaluation, and Treatment of High Blood Cholesterol in Adults (Adult Treatment Panel III). Third Report of the National Cholesterol Education Program (NCEP) Expert Panel on Detection, Evaluation, and Treatment of High Blood Cholesterol in Adults (Adult Treatment Panel III) final report. Circulation.

[18] Wang K., Li M., Hakonarson H. (2010). ANNOVAR: Functional annotation of genetic variants from high-throughput sequencing data. Nucleic Acids Res..

[19] Kircher M., Witten D.M., Jain P., O’Roak B.J., Cooper G.M., Shendure J. (2014). A general framework for estimating the relative pathogenicity of human genetic variants. Nat. Genet..

[20] Quang D., Chen Y., Xie X. (2015). DANN: A deep learning approach for annotating the pathogenicity of genetic variants. Bioinformatics.

[21] Boyle A.P., Hong E.L., Hariharan M., Cheng Y., Schaub M.A., Kasowski M., Karczewski K.J., Park J., Hitz B.C., Weng S. (2012). Annotation of functional variation in personal genomes using RegulomeDB. Genome Res..

[22] Miller M., Rhyne J., Chen H., Beach V., Ericson R., Luthra K., Dwivedi M., Misra A. (2007). APOC3 promoter polymorphisms C-482T and T-455C are associated with the metabolic syndrome. Arch. Med. Res..

[23] Wu Y., Yu Y., Zhao T., Wang S., Fu Y., Qi Y., Yang G., Yao W., Su Y., Ma Y. (2016). Interactions of Environmental Factors and APOA1-APOC3-APOA4-APOA5 Gene Cluster Gene Polymorphisms with Metabolic Syndrome. PLoS ONE.

[24] Castellano-Castillo D., Moreno-Indias I., Fernandez-Garcia J.C., Alcaide-Torres J., Moreno-Santos I., Ocana L., Gluckman E., Tinahones F., Queipo-Ortuno M.I., Cardona F. (2018). Adipose Tissue LPL Methylation is Associated with Triglyceride Concentrations in the Metabolic Syndrome. Clin. Chem..

[25] Zahedi A.S., Akbarzadeh M., Sedaghati-Khayat B., Seyedhamzehzadeh A., Daneshpour M.S. (2021). GCKR common functional polymorphisms are associated with metabolic syndrome and its components: A 10-year retrospective cohort study in Iranian adults. Diabetol. Metab. Syndr..

[26] Feng L., Sun Y., Liu F., Wang C., Zhang C., Liu J., Jiang L. (2022). Clinical features and functions of a novel Lpl mutation C.986A>C (p.Y329S) in patient with hypertriglyceridemia. Curr. Res. Transl. Med..

[27] Wong S.K., Ramli F.F., Ali A., Ibrahim N. (2022). Genetics of Cholesterol-Related Genes in Metabolic Syndrome: A Review of Current Evidence. Biomedicines.

[28] Shen M., Jiang L., Liu H., Dai H., Jiang H., Qian Y., Wang Z., Zheng S., Chen H., Yang T. (2023). Interaction between the GCKR rs1260326 variant and serum HDL cholesterol contributes to HOMA-beta and ISI(Matusda) in the middle-aged T2D individuals. J. Hum. Genet..

[29] Lee H.J., Jang H.B., Kim H.J., Ahn Y., Hong K.W., Cho S.B., Kang J.H., Park S.I. (2015). The dietary monounsaturated to saturated fatty acid ratio modulates the genetic effects of GCKR on serum lipid levels in children. Clin. Chim. Acta.

[30] Xu M., Lv X., Xie L., Huang X., Huang Y., Chen Y., Peng K., Wang P., Wang W., Qi L. (2016). Discrete associations of the GCKR variant with metabolic risk in a Chinese population: Longitudinal change analysis. Diabetologia.

[31] Akbarzadeh M., Alipour N., Moheimani H., Zahedi A.S., Hosseini-Esfahani F., Lanjanian H., Azizi F., Daneshpour M.S. (2022). Evaluating machine learning-powered classification algorithms which utilize variants in the GCKR gene to predict metabolic syndrome: Tehran Cardio-metabolic Genetics Study. J. Transl. Med..

[32] Simons P., Simons N., Stehouwer C.D.A., Schalkwijk C.G., Schaper N.C., Brouwers M. (2018). Association of common gene variants in glucokinase regulatory protein with cardiorenal disease: A systematic review and meta-analysis. PLoS ONE.

[33] Lian J., Guo J., Chen Z., Jiang Q., Ye H., Huang X., Yang X., Ba Y., Zhou J., Duan S. (2013). Positive association between GCKR rs780093 polymorphism and coronary heart disease in the aged Han Chinese. Dis. Markers.

[34] Ma L., Brautbar A., Boerwinkle E., Sing C.F., Clark A.G., Keinan A. (2012). Knowledge-driven analysis identifies a gene-gene interaction affecting high-density lipoprotein cholesterol levels in multi-ethnic populations. PLoS Genet..

[35] Al Madhoun A. (2024). Glucokinase regulatory protein rs780094 polymorphism is associated with type 2 diabetes mellitus, dyslipidemia, non-alcoholic fatty liver disease, and nephropathy. World J. Diabetes.

[36] Liu Y.Y., Wan Q. (2023). Relationship between GCKR gene rs780094 polymorphism and type 2 diabetes with albuminuria. World J. Diabetes.

[37] Guan F., Niu Y., Zhang T., Liu S., Ma L., Qi T., Feng J., Zuo H., Li G., Liu X. (2016). Two-stage association study to identify the genetic susceptibility of a novel common variant of rs2075290 in ZPR1 to type 2 diabetes. Sci. Rep..

[38] Kong X., Zhang X., Xing X., Zhang B., Hong J., Yang W. (2015). The Association of Type 2 Diabetes Loci Identified in Genome-Wide Association Studies with Metabolic Syndrome and Its Components in a Chinese Population with Type 2 Diabetes. PLoS ONE.

[39] Shang X., Song J., Liu F., Ma J., Wang H. (2014). Association between rs780094 polymorphism in GCKR and plasma lipid levels in children and adolescents. Zhonghua Liu Xing Bing Xue Za Zhi.

[40] Onuma H., Tabara Y., Kawamoto R., Shimizu I., Kawamura R., Takata Y., Nishida W., Ohashi J., Miki T., Kohara K. (2010). The GCKR rs780094 polymorphism is associated with susceptibility of type 2 diabetes, reduced fasting plasma glucose levels, increased triglycerides levels and lower HOMA-IR in Japanese population. J. Hum. Genet..

[41] Bi M., Kao W.H., Boerwinkle E., Hoogeveen R.C., Rasmussen-Torvik L.J., Astor B.C., North K.E., Coresh J., Köttgen A. (2010). Association of rs780094 in GCKR with metabolic traits and incident diabetes and cardiovascular disease: The ARIC Study. PLoS ONE.

[42] Go M.J., Hwang J.Y., Kim D.J., Lee H.J., Jang H.B., Park K.H., Song J., Lee J.Y. (2012). Effect of genetic predisposition on blood lipid traits using cumulative risk assessment in the korean population. Genom. Inform..

[43] Rafiq S., Venkata K.K., Gupta V., Vinay D.G., Spurgeon C.J., Parameshwaran S., Madana S.N., Kinra S., Bowen L., Timpson N.J. (2012). Evaluation of seven common lipid associated loci in a large Indian sib pair study. Lipids Health Dis..

[44] Baik I., Lee S., Kim S.H., Shin C. (2013). A lipoprotein lipase gene polymorphism interacts with consumption of alcohol and unsaturated fat to modulate serum HDL-cholesterol concentrations. J. Nutr..

[45] Angelakopoulou A., Shah T., Sofat R., Shah S., Berry D.J., Cooper J., Palmen J., Tzoulaki I., Wong A., Jefferis B.J. (2012). Comparative analysis of genome-wide association studies signals for lipids, diabetes, and coronary heart disease: Cardiovascular Biomarker Genetics Collaboration. Eur. Heart J..

[46] Ma Y., Tucker K.L., Smith C.E., Lee Y.C., Huang T., Richardson K., Parnell L.D., Lai C.Q., Young K.L., Justice A.E. (2014). Lipoprotein lipase variants interact with polyunsaturated fatty acids for obesity traits in women: Replication in two populations. Nutr. Metab. Cardiovasc. Dis..

[47] Paththinige C.S., Sirisena N.D., Dissanayake V. (2017). Genetic determinants of inherited susceptibility to hypercholesterolemia—A comprehensive literature review. Lipids Health Dis..

[48] Kwak J., Hong G., Lee K.J., Kim C.G., Shin D. (2023). Effect of the Interaction between Seaweed Intake and LPL Polymorphisms on Metabolic Syndrome in Middle-Aged Korean Adults. Nutrients.

[49] Walia G.K., Panniyammakal J., Agarwal T., Jalal R., Gupta R., Ramakrishnan L., Tandon N., Roy A., Krishnan A., Prabhakaran D. (2023). Evaluation of genetic variants related to lipid levels among the North Indian population. Front. Genet..

[50] Wallace C., Newhouse S.J., Braund P., Zhang F., Tobin M., Falchi M., Ahmadi K., Dobson R.J., Marcano A.C., Hajat C. (2008). Genome-wide association study identifies genes for biomarkers of cardiovascular disease: Serum urate and dyslipidemia. Am. J. Hum. Genet..

[51] Franzago M., Di Nicola M., Fraticelli F., Marchioni M., Stuppia L., Vitacolonna E. (2022). Nutrigenetic variants and response to diet/lifestyle intervention in obese subjects: A pilot study. Acta Diabetol..

[52] Ho C.Y., Lee J.I., Huang S.P., Chen S.C., Geng J.H. (2023). A Genome-Wide Association Study of Metabolic Syndrome in the Taiwanese Population. Nutrients.

[53] Liu Y., Zhou D., Zhang Z., Song Y., Zhang D., Zhao T., Chen Z., Sun Y., Zhang D., Yang Y. (2011). Effects of genetic variants on lipid parameters and dyslipidemia in a Chinese population. J. Lipid Res..

[54] Malek S.H., Al-Serri A.E., Al-Bustan S.A. (2021). Genetic association of LPL rs326 with BMI among the Kuwaiti population. Cardiovasc. Endocrinol. Metab..

[55] Mitrokhin V., Nikitin A., Brovkina O., Khodyrev D., Zotov A., Vachrushev N., Dragunov D., Shim A., Mladenov M., Kamkin A. (2018). Association between IL-18/18R gene polymorphisms and coronary artery disease: Influence of IL-18/18R genetic variants on cytokine expression. J. Inflamm. Res..

[56] Tang W., Apostol G., Schreiner P.J., Jacobs D.R., Boerwinkle E., Fornage M. (2010). Associations of lipoprotein lipase gene polymorphisms with longitudinal plasma lipid trends in young adults: The Coronary Artery Risk Development in Young Adults (CARDIA) study. Circ. Cardiovasc. Genet..

[57] Zhu X.C., Lin J., Wang Q., Liu H., Qiu L., Fang D.Z. (2014). Associations of lipoprotein lipase gene rs326 with changes of lipid profiles after a high-carbohydrate and low-fat diet in healthy Chinese Han youth. Int. J. Environ. Res. Public Health.

[58] Johansen C.T., Kathiresan S., Hegele R.A. (2011). Genetic determinants of plasma triglycerides. J. Lipid Res..

[59] Johansen C.T., Wang J., Lanktree M.B., Cao H., McIntyre A.D., Ban M.R., Martins R.A., Kennedy B.A., Hassell R.G., Visser M.E. (2010). Excess of rare variants in genes identified by genome-wide association study of hypertriglyceridemia. Nat. Genet..

[60] Gombojav B., Lee S.J., Kho M., Song Y.M., Lee K., Sung J. (2016). Multiple susceptibility loci at chromosome 11q23.3 are associated with plasma triglyceride in East Asians. J. Lipid Res..

[61] Liu Y., Ordovas J.M., Gao G., Province M., Straka R.J., Tsai M.Y., Lai C.Q., Zhang K., Borecki I., Hixson J.E. (2009). Pharmacogenetic association of the APOA1/C3/A4/A5 gene cluster and lipid responses to fenofibrate: The genetics of lipid-lowering drugs and diet network study. Pharmacogenet. Genom..

[62] Ruixing Y., Yiyang L., Meng L., Kela L., Xingjiang L., Lin Z., Wanying L., Jinzhen W., Dezhai Y., Weixiong L. (2010). Interactions of the apolipoprotein C-III 3238C>G polymorphism and alcohol consumption on serum triglyceride levels. Lipids Health Dis..

[63] Adams J.N., Raffield L.M., Freedman B.I., Langefeld C.D., Ng M.C., Carr J.J., Cox A.J., Bowden D.W. (2014). Analysis of common and coding variants with cardiovascular disease in the Diabetes Heart Study. Cardiovasc. Diabetol..

[64] Hosseini-Esfahani F., Mirmiran P., Daneshpour M.S., Mehrabi Y., Hedayati M., Zarkesh M., Azizi F. (2014). Western dietary pattern interaction with APOC3 polymorphism in the risk of metabolic syndrome: Tehran Lipid and Glucose Study. J. Nutr. Nutr..

[65] Hosseini-Esfahani F., Mirmiran P., Daneshpour M.S., Mehrabi Y., Hedayati M., Soheilian-Khorzoghi M., Azizi F. (2015). Dietary patterns interact with APOA1/APOC3 polymorphisms to alter the risk of the metabolic syndrome: The Tehran Lipid and Glucose Study. Br. J. Nutr..

[66] Ou H.J., Huang G., Liu W., Ma X.L., Wei Y., Zhou T., Pan Z.M. (2015). Relationship of the APOA5/A4/C3/A1 gene cluster and APOB gene polymorphisms with dyslipidemia. Genet. Mol. Res..

[67] Song Y., Zhu L., Richa M., Li P., Yang Y., Li S. (2015). Associations of the APOC3 rs5128 polymorphism with plasma APOC3 and lipid levels: A meta-analysis. Lipids Health Dis..

[68] Hosseini-Esfahani F., Mirmiran P., Daneshpour M.S., Mottaghi A., Azizi F. (2017). The Effect of Interactions of Single Nucleotide Polymorphisms of APOA1/APOC3 with Food Group Intakes on the Risk of Metabolic Syndrome. Avicenna J. Med. Biotechnol..

[69] Goyal S., Tanigawa Y., Zhang W., Chai J.F., Almeida M., Sim X., Lerner M., Chainakul J., Ramiu J.G., Seraphin C. (2021). APOC3 genetic variation, serum triglycerides, and risk of coronary artery disease in Asian Indians, Europeans, and other ethnic groups. Lipids Health Dis..

[70] Chowdhary R., Masarkar N., Khadanga S. (2022). Polymorphism in Apolipoprotein C3 (APOC3) and Fatty Acid-Binding Proteins (FABP2) Genes in Nondiabetic Dyslipidemic Patients: A Tertiary Care Hospital-Based Pilot Study. J. Lab. Physicians.

[71] Cha S., Yu H., Park A.Y., Song K.H. (2014). Effects of apolipoprotein A5 haplotypes on the ratio of triglyceride to high-density lipoprotein cholesterol and the risk for metabolic syndrome in Koreans. Lipids Health Dis..

[72] Gorre M., Rayabarapu P., Battini S.R., Irgam K., Battini M.R. (2022). Analysis of 61 SNPs from the CAD specific genomic loci reveals unique set of SNPs as significant markers in the Southern Indian population of Hyderabad. BMC Cardiovasc. Disord..

[73] Lee J.S., Cheong H.S., Shin H.D. (2018). Prediction of cholesterol ratios within a Korean population. R. Soc. Open Sci..

[74] Pranavchand R., Kumar A.S., Reddy B.M. (2017). Genetic determinants of clinical heterogeneity of the coronary artery disease in the population of Hyderabad, India. Hum. Genom..

[75] Kim T., Park A.Y., Baek Y., Cha S. (2017). Genome-Wide Association Study Reveals Four Loci for Lipid Ratios in the Korean Population and the Constitutional Subgroup. PLoS ONE.

[76] Pranav Chand R., Kumar A.S., Anuj K., Vishnupriya S., Mohan Reddy B. (2016). Distinct Patterns of Association of Variants at 11q23.3 Chromosomal Region with Coronary Artery Disease and Dyslipidemia in the Population of Andhra Pradesh, India. PLoS ONE.

[77] Fu Q., Tang X., Chen J., Su L., Zhang M., Wang L., Jing J., Zhou L. (2015). Effects of Polymorphisms in APOA4-APOA5-ZNF259-BUD13 Gene Cluster on Plasma Levels of Triglycerides and Risk of Coronary Heart Disease in a Chinese Han Population. PLoS ONE.

[78] Ellakwa D.E., Amr K.S., Zaki M.E., Refeat M., Banksle H.M. (2024). Zinc finger 259 gene polymorphisms in Egyptian patients with metabolic syndrome and its association with dyslipidemia. Ir. J. Med. Sci..

[79] Mirhafez S.R., Avan A., Pasdar A., Khatamianfar S., Hosseinzadeh L., Ganjali S., Movahedi A., Pirhoushiaran M., Mellado V.G., Rosace D. (2016). Zinc Finger 259 Gene Polymorphism rs964184 is Associated with Serum Triglyceride Levels and Metabolic Syndrome. Int. J. Mol. Cell Med..

[80] Wu Y., Yu S., Wang S., Shi J., Xu Z., Zhang Q., Fu Y., Qi Y., Liu J., Fu R. (2015). Zinc Finger Protein 259 (ZNF259) Polymorphisms are Associated with the Risk of Metabolic Syndrome in a Han Chinese Population. Clin. Lab..

[81] Ueyama C., Horibe H., Yamase Y., Fujimaki T., Oguri M., Kato K., Arai M., Watanabe S., Murohara T., Yamada Y. (2015). Association of FURIN and ZPR1 polymorphisms with metabolic syndrome. Biomed. Rep..

[82] Liu M., Jin H.S., Park S. (2020). Protein and fat intake interacts with the haplotype of PTPN11_rs11066325, RPH3A_rs886477, and OAS3_rs2072134 to modulate serum HDL concentrations in middle-aged people. Clin. Nutr..

[83] Park S., Liu M., Kang S. (2018). Alcohol Intake Interacts with CDKAL1, HHEX, and OAS3 Genetic Variants, Associated with the Risk of Type 2 Diabetes by Lowering Insulin Secretion in Korean Adults. Alcohol. Clin. Exp. Res..

[84] Heo S.G., Hwang J.Y., Uhmn S., Go M.J., Oh B., Lee J.Y., Park J.W. (2014). Male-specific genetic effect on hypertension and metabolic disorders. Hum. Genet..

[85] Cho S.B., Jang J. (2021). A Genome-Wide Association Study of a Korean Population Identifies Genetic Susceptibility to Hypertension Based on Sex-Specific Differences. Genes.

[86] Kim J., Oh B., Lim J.E., Kim M.K. (2016). No Interaction with Alcohol Consumption, but Independent Effect of C12orf51 (HECTD4) on Type 2 Diabetes Mellitus in Korean Adults Aged 40-69 Years: The KoGES_Ansan and Ansung Study. PLoS ONE.

[87] Silbernagel G., Scharnagl H., Kleber M.E., Delgado G., Stojakovic T., Laaksonen R., Erdmann J., Rankinen T., Bouchard C., Landmesser U. (2019). LDL triglycerides, hepatic lipase activity, and coronary artery disease: An epidemiologic and Mendelian randomization study. Atherosclerosis.

[88] Yuan M.Z., Han R.A., Zhang C.X., Chen Y.X. (2018). Association of Genes in the High-Density Lipoprotein Metabolic Pathway with Polypoidal Choroidal Vasculopathy in Asian Population: A Systematic Review and Meta-Analysis. J. Ophthalmol..

[89] Ambroselli D., Masciulli F., Romano E., Catanzaro G., Besharat Z.M., Massari M.C., Ferretti E., Migliaccio S., Izzo L., Ritieni A. (2023). New Advances in Metabolic Syndrome, from Prevention to Treatment: The Role of Diet and Food. Nutrients.

[90] Wu Q., Li J., Sun X., He D., Cheng Z., Li J., Zhang X., Xie Y., Zhu Y., Lai M. (2021). Multi-stage metabolomics and genetic analyses identified metabolite biomarkers of metabolic syndrome and their genetic determinants. EBioMedicine.

[91] Reilly D., Hao K., Jensen M.K., Girman C.J., Rimm E.B. (2013). Use of systems biology approaches to analysis of genome-wide association studies of myocardial infarction and blood cholesterol in the nurses’ health study and health professionals’ follow-up study. PLoS ONE.

[92] Teng M.S., Wu S., Hsu L.A., Tzeng I.S., Chou H.H., Su C.W., Ko Y.L. (2019). Pleiotropic association of LIPC variants with lipid and urinary 8-hydroxy deoxyguanosine levels in a Taiwanese population. Lipids Health Dis..

[93] Teng M.S., Wu S., Er L.K., Hsu L.A., Chou H.H., Ko Y.L. (2018). LIPC variants as genetic determinants of adiposity status, visceral adiposity indicators, and triglyceride-glucose (TyG) index-related parameters mediated by serum triglyceride levels. Diabetol. Metab. Syndr..

[94] Khounphinith E., Yin R.X., Qiu L., Zhang F.H., Yan R.Q., Lu L., Su Y. (2018). Association of the LIPC rs1532085 SNP and serum lipid traits in the Chinese Maonan and Han populations. Int. J. Clin. Exp. Pathol..

[95] Cui M., Li W., Ma L., Ping F., Liu J., Wu X., Mao J., Wang X., Nie M. (2017). HDL-cholesterol concentration in pregnant Chinese Han women of late second trimester associated with genetic variants in CETP, ABCA1, APOC3, and GALNT2. Oncotarget.

[96] Meng Q., Huang L., Sun Y., Bai Y., Wang B., Yu W., Zhao M., Li X. (2015). Effect of High-Density Lipoprotein Metabolic Pathway Gene Variations and Risk Factors on Neovascular Age-Related Macular Degeneration and Polypoidal Choroidal Vasculopathy in China. PLoS ONE.

[97] Ma L., Ballantyne C., Brautbar A., Keinan A. (2014). Analysis of multiple association studies provides evidence of an expression QTL hub in gene-gene interaction network affecting HDL cholesterol levels. PLoS ONE.

[98] Simmons C.R., Zou F., Younkin S.G., Estus S. (2011). Evaluation of the global association between cholesterol-associated polymorphisms and Alzheimer’s disease suggests a role for rs3846662 and HMGCR splicing in disease risk. Mol. Neurodegener..

[99] Santoro N., Chen L., Todd J., Divers J., Shah A.S., Gidding S.S., Burke B., Haymond M., Lange L., Marcovina S. (2021). Genome-wide Association Study of Lipid Traits in Youth with Type 2 Diabetes. J. Endocr. Soc..

[100] Prasad G., Bandesh K., Giri A.K., Kauser Y., Chanda P., Parekatt V., Mathur S., Madhu S.V., Venkatesh P., Bhansali A. (2019). Genome-Wide Association Study of Metabolic Syndrome Reveals Primary Genetic Variants at CETP Locus in Indians. Biomolecules.

[101] Blackburn N.B., Porto A., Peralta J.M., Blangero J. (2018). Heritability and genetic associations of triglyceride and HDL-C levels using pedigree-based and empirical kinships. BMC Proc..

[102] Raina J.K., Sharma M., Panjaliya R.K., Dogra V., Bakaya A., Kumar P. (2020). Association of ESR1 (rs2234693 and rs9340799), CETP (rs708272), MTHFR (rs1801133 and rs2274976) and MS (rs185087) polymorphisms with Coronary Artery Disease (CAD). BMC Cardiovasc. Disord..

[103] Nagrani R., Foraita R., Gianfagna F., Iacoviello L., Marild S., Michels N., Molnár D., Moreno L., Russo P., Veidebaum T. (2020). Common genetic variation in obesity, lipid transfer genes and risk of Metabolic Syndrome: Results from IDEFICS/I.Family study and meta-analysis. Sci. Rep..

[104] El-Lebedy D. (2018). Interaction between endothelial nitric oxide synthase rs1799983, cholesteryl ester-transfer protein rs708272 and angiopoietin-like protein 8 rs2278426 gene variants highly elevates the risk of type 2 diabetes mellitus and cardiovascular disease. Cardiovasc. Diabetol..

[105] Maroufi N.F., Farzaneh K., Alibabrdel M., Zarei L., Cheraghi O., Soltani S., Montazersaheb S., Akbarzadeh M., Nouri M. (2016). Taq1B Polymorphism of Cholesteryl Ester Transfer Protein (CETP) and Its Effects on the Serum Lipid Levels in Metabolic Syndrome Patients. Biochem. Genet..

[106] Cahua-Pablo J., Cruz M., Méndez-Palacios A., Antúnez-Ortiz D.L., Vences-Velázquez A., del Carmen Alarcón-Romero L., Parra E.J., Tello-Flores V.A., Leyva-Vázquez M.A., Valladares-Salgado A. (2015). Polymorphisms in the LPL and CETP Genes and Haplotype in the ESR1 Gene Are Associated with Metabolic Syndrome in Women from Southwestern Mexico. Int. J. Mol. Sci..

[107] Dullaart R.P., Sluiter W.J. (2008). Common variation in the CETP gene and the implications for cardiovascular disease and its treatment: An updated analysis. Pharmacogenomics.

[108] Yu X., Duan R., Niu Y., Ding Y., Zhu B., Xu W., Bai W., Wu Y., Yu Y., Du H. (2021). Association of GCKR Gene Polymorphisms with Metabolic Syndrome in a Han Population from Northeast China. Clin. Lab..

[109] Franssen R., Monajemi H., Stroes E.S., Kastelein J.J. (2011). Obesity and dyslipidemia. Med. Clin. N. Am..

[110] Kawashima R.L., Medh J.D. (2014). Down-regulation of lipoprotein lipase increases ABCA1-mediated cholesterol efflux in THP-1 macrophages. Biochem. Biophys. Res. Commun..

[111] Vishram J.K., Hansen T.W., Torp-Pedersen C., Madsbad S., Jorgensen T., Fenger M., Lyngbaek S., Jeppesen J. (2016). Relationship Between Two Common Lipoprotein Lipase Variants and the Metabolic Syndrome and Its Individual Components. Metab. Syndr. Relat. Disord..

[112] Liao Y.H., Er L.K., Wu S., Ko Y.L., Teng M.S. (2021). Functional Haplotype of LIPC Induces Triglyceride-Mediated Suppression of HDL-C Levels According to Genome-Wide Association Studies. Genes.

[113] Lim G.B. (2022). LIPC variant in combined hypocholesterolaemia. Nat. Rev. Cardiol..

[114] Ozsait B., Komurcu Bayrak E., Poda M., Can G., Hergenc G., Onat A., Humphries S.E., Erginel Unaltuna N. (2008). CETP TaqIB polymorphism in Turkish adults: Association with dyslipidemia and metabolic syndrome. Anadolu Kardiyol. Derg..

[115] Girona J., Ibarretxe D., Plana N., Guaita-Esteruelas S., Amigo N., Heras M., Masana L. (2016). Circulating PCSK9 levels and CETP plasma activity are independently associated in patients with metabolic diseases. Cardiovasc. Diabetol..

[116] Garcia-Rios A., Alcala-Diaz J.F., Gomez-Delgado F., Delgado-Lista J., Marin C., Leon-Acuna A., Camargo A., Rodriguez-Cantalejo F., Blanco-Rojo R., Quintana-Navarro G. (2018). Beneficial effect of CETP gene polymorphism in combination with a Mediterranean diet influencing lipid metabolism in metabolic syndrome patients: CORDIOPREV study. Clin. Nutr..

[117] Tarasco E., Boyle C.N., Pellegrini G., Arnold M., Steiner R., Hornemann T., Nasias D., Kardassis D., Whiting L., Lutz T.A. (2019). Body weight-dependent and independent improvement in lipid metabolism after Roux-en-Y gastric bypass in ApoE*3Leiden.CETP mice. Int. J. Obes..

[118] Alberti K.G., Zimmet P., Shaw J. (2006). Metabolic syndrome—A new world-wide definition. A Consensus Statement from the International Diabetes Federation. Diabet. Med..

[119] Barkas F., Elisaf M., Liberopoulos E., Liontos A., Rizos E.C. (2016). High triglyceride levels alter the correlation of apolipoprotein B with low- and non-high-density lipoprotein cholesterol mostly in individuals with diabetes or metabolic syndrome. Atherosclerosis.

[120] Pantoja-Torres B., Toro-Huamanchumo C.J., Urrunaga-Pastor D., Guarnizo-Poma M., Lazaro-Alcantara H., Paico-Palacios S., Del Carmen Ranilla-Seguin V., Benites-Zapata V.A. (2019). High triglycerides to HDL-cholesterol ratio is associated with insulin resistance in normal-weight healthy adults. Diabetes Metab. Syndr..

[121] Schrezenmeir J., Fenselau S., Keppler I., Abel J., Orth B., Laue C., Sturmer W., Fauth U., Halmagyi M., Marz W. (1997). Postprandial triglyceride high response and the metabolic syndrome. Ann. N. Y. Acad. Sci..

[122] Denimal D., Monier S., Bouillet B., Verges B., Duvillard L. (2023). High-Density Lipoprotein Alterations in Type 2 Diabetes and Obesity. Metabolites.

[123] Ji J., Watts G.F., Johnson A.G., Chan D.C., Ooi E.M., Rye K.A., Serone A.P., Barrett P.H. (2006). High-density lipoprotein (HDL) transport in the metabolic syndrome: Application of a new model for HDL particle kinetics. J. Clin. Endocrinol. Metab..

[124] Vollenweider P., von Eckardstein A., Widmann C. (2015). HDLs, diabetes, and metabolic syndrome. Handbook of Experimental Pharmacology.

[125] Lee H.C., Shin S.J., Huang J.K., Lin M.Y., Lin Y.H., Ke L.Y., Jiang H.J., Tsai W.C., Chao M.F., Lin Y.H. (2020). The role of postprandial very-low-density lipoprotein in the development of atrial remodeling in metabolic syndrome. Lipids Health Dis..

[126] Soderlund S., Watanabe H., Ehnholm C., Jauhiainen M., Taskinen M.R. (2010). Increased apolipoprotein E level and reduced high-density lipoprotein mean particle size associate with low high-density lipoprotein cholesterol and features of metabolic syndrome. Metabolism.

[127] Srisawasdi P., Vanavanan S., Rochanawutanon M., Kruthkul K., Kotani K., Kroll M.H. (2015). Small-dense LDL/large-buoyant LDL ratio associates with the metabolic syndrome. Clin. Biochem..

[128] Ooi E.M., Ng T.W., Chan D.C., Watts G.F. (2009). Plasma markers of cholesterol homeostasis in metabolic syndrome subjects with or without type-2 diabetes. Diabetes Res. Clin. Pract..

[129] Ouimet M. (2013). Autophagy in obesity and atherosclerosis: Interrelationships between cholesterol homeostasis, lipoprotein metabolism and autophagy in macrophages and other systems. Biochim. Biophys. Acta.

[130] Taverne F., Richard C., Couture P., Lamarche B. (2013). Abdominal obesity, insulin resistance, metabolic syndrome and cholesterol homeostasis. PharmaNutrition.

[131] Yamaoka-Tojo M., Tojo T., Izumi T. (2008). Beyond cholesterol lowering: Pleiotropic effects of bile acid binding resins against cardiovascular disease risk factors in patients with metabolic syndrome. Curr. Vasc. Pharmacol..

[132] Pownall H.J., Rosales C., Gillard B.K., Gotto A.M. (2021). High-density lipoproteins, reverse cholesterol transport and atherogenesis. Nat. Rev. Cardiol..

[133] Vasudevan M., Tchoua U., Gillard B.K., Jones P.H., Ballantyne C.M., Pownall H.J. (2013). Modest diet-induced weight loss reduces macrophage cholesterol efflux to plasma of patients with metabolic syndrome. J. Clin. Lipidol..

[134] von Eckardstein A. (2020). LDL Contributes to Reverse Cholesterol Transport. Circ. Res..

[135] Wang H.H., Garruti G., Liu M., Portincasa P., Wang D.Q. (2017). Cholesterol and Lipoprotein Metabolism and Atherosclerosis: Recent Advances in reverse Cholesterol Transport. Ann. Hepatol..

[136] Perez-Martinez P., Alcala-Diaz J.F., Delgado-Lista J., Garcia-Rios A., Gomez-Delgado F., Marin-Hinojosa C., Rodriguez-Cantalejo F., Delgado-Casado N., Perez-Caballero A.I., Fuentes-Jimenez F.J. (2014). Metabolic phenotypes of obesity influence triglyceride and inflammation homoeostasis. Eur. J. Clin. Investig..

[137] Ramírez C.M., Goedeke L., Fernández-Hernando C. (2011). “Micromanaging” metabolic syndrome. Cell Cycle.

[138] Lupattelli G., De Vuono S., Mannarino E. (2011). Patterns of cholesterol metabolism: Pathophysiological and therapeutic implications for dyslipidemias and the metabolic syndrome. Nutr. Metab. Cardiovasc. Dis..

[139] Brunham L.R., Kruit J.K., Verchere C.B., Hayden M.R. (2008). Cholesterol in islet dysfunction and type 2 diabetes. J. Clin. Investig..

[140] Povel C.M., Boer J.M., Reiling E., Feskens E.J. (2011). Genetic variants and the metabolic syndrome: A systematic review. Obes. Rev..

[141] Fallah M.S., Sedaghatikhayat B., Guity K., Akbari F., Azizi F., Daneshpour M.S. (2016). The Relation between Metabolic Syndrome Risk Factors and Genetic Variations of Apolipoprotein V in Relation with Serum Triglyceride and HDL-C Level. Arch. Iran. Med..

[142] Yamada Y., Kato K., Oguri M., Horibe H., Fujimaki T., Yasukochi Y., Takeuchi I., Sakuma J. (2018). Identification of four genes as novel susceptibility loci for early-onset type 2 diabetes mellitus, metabolic syndrome, or hyperuricemia. Biomed. Rep..

[143] Hechmi M., Dallali H., Gharbi M., Jmel H., Fassatoui M., Ben Halima Y., Bahri S., Bahlous A., Abid A., Jamoussi H. (2020). Association of rs662799 variant and APOA5 gene haplotypes with metabolic syndrome and its components: A meta-analysis in North Africa. Biosci. Rep..

[144] Hsu M.C., Chang C.S., Lee K.T., Sun H.Y., Tsai Y.S., Kuo P.H., Young K.C., Wu C.H. (2013). Central obesity in males affected by a dyslipidemia-associated genetic polymorphism on APOA1/C3/A4/A5 gene cluster. Nutr. Diabetes.

[145] Son K.Y., Son H.Y., Chae J., Hwang J., Jang S., Yun J.M., Cho B., Park J.H., Kim J.I. (2015). Genetic association of APOA5 and APOE with metabolic syndrome and their interaction with health-related behavior in Korean men. Lipids Health Dis..

[146] Kim S.-S., Park S., Jin H.-S. (2020). Replicated Association Study for Metabolic Syndrome of the Gene Cluster in Chromosome 11q23. 3. Biomed. Sci. Lett..

[147] Moon S., Kim Y.J., Han S., Hwang M.Y., Shin D.M., Park M.Y., Lu Y., Yoon K., Jang H.M., Kim Y.K. (2019). The Korea Biobank Array: Design and Identification of Coding Variants Associated with Blood Biochemical Traits. Sci. Rep..

[148] Purcell S., Neale B., Todd-Brown K., Thomas L., Ferreira M.A., Bender D., Maller J., Sklar P., de Bakker P.I., Daly M.J. (2007). PLINK: A tool set for whole-genome association and population-based linkage analyses. Am. J. Hum. Genet..

[149] Dennis G., Sherman B.T., Hosack D.A., Yang J., Gao W., Lane H.C., Lempicki R.A. (2003). DAVID: Database for Annotation, Visualization, and Integrated Discovery. Genome Biol..

