# Supplementary material for: Multivariate Genome-Wide Association Analysis Identifies Genetic Susceptibility Loci for Metabolic Syndrome in the Korean Genome and Epidemiology Study
Source: Int J Mol Sci. 2026 Sep 9;27(18):8041. doi: 10.3390/ijms27188041 (PMC13606617; doi:10.3390/ijms27188041)
Supplement: Supplementary file 1 [file ijms-27-08041-s001.zip › ijms-4495876-supplementary.pdf]

Supplementary Methods, Tables and Figures

Multivariate Genome-Wide Association Analysis Identifies Genetic Susceptibility Loci for Metabolic Syndrome in the Korean Genome and Epidemiology Study

Dasom Kim [1,†], Junho Cha [2,†] and Sungkyoung Choi [1,2,*]

1 Department of Mathematical Data Science, College of Computing, Hanyang University, 55 Hanyang-Daehak-ro, Sangnok-gu, Ansan 15588, Republic of Korea; ektha20@hanyang.ac.kr
2 Department of Applied Artificial Intelligence, College of Computing, Hanyang University, 55 Hanyang-Daehak-ro, Sangnok-gu, Ansan 15588, Republic of Korea; chajunho822@hanyang.ac.kr
† The first two authors should be regarded as joint First Authors.
* Correspondence: day0413@hanyang.ac.kr; Tel.: +82-31-400-5465

Text S1. Supplementary Methods

This section provides the detailed statistical models and analysis parameters underlying the multiple-trait analyses (MPAT and GEMMA) and the supporting annotation and enrichment procedures. Notation: for a given single-nucleotide polymorphism (SNP), $K = 6$ continuous MetS-related traits (waist circumference, systolic and diastolic blood pressure, log-triglycerides, log-fasting plasma glucose, and negative-log HDL-C) were analyzed jointly.

S1.1. Trait definition and transformation

MetS was defined by the NCEP ATP III criteria (>= 3 of 5 components). For the multiple-trait analyses, the five diagnostic components were represented by six continuous MetS-related traits: waist circumference (WC); systolic and diastolic blood pressure (SBP and DBP), modeled separately; log(triglycerides) [log(TG)]; log(fasting plasma glucose) [log(FPG)]; and negative-log(high-density lipoprotein cholesterol) [-log(HDL-C)]. TG, FPG, and HDL-C were transformed to reduce right-skewness and stabilize variance; the negative sign for HDL-C aligns its direction of risk with the other traits. All traits were adjusted for age, sex, and (discovery cohort only) recruitment area.

S1.2. Multiple-Trait Association Test (MPAT)

MPAT jointly tests the association of a variant with the K correlated traits from the inivariate (per-trait) summary statistics, following Liu and Lin (2018). For a given SNP, let $\boldsymbol{Z} = (Z_1, ..., Z_K)^T$ be the vector of univariate Wald ($\boldsymbol{Z}$) statistics form the K single-trait analyses. A key result of Liu and Lin is that, under the null, the correlation matrix of $\boldsymbol{Z}$ equals the residual between-trait correlation matrix $\boldsymbol{\Sigma}$ and does not depend on genotype; $\boldsymbol{\Sigma}$ is therefore estimated consistently from the sample correlation of the genome-wide $\boldsymbol{Z}$ statistics across null SNPs. Under the null, $\boldsymbol{Z}$ is approximately $N(0, \boldsymbol{\Sigma})$. MPAT combines two complementary score statistics, derived from a linear mixed model for $\boldsymbol{Z}$, that capture homogeneous and heterogeneous genetic effects.

SUM (homogeneous effects):

Testing a shared common effect ($\mu_k = \mu_0\boldsymbol{J}$, with $\boldsymbol{J} = (1, ..., 1)^T$) gives the score statistic

$$\mathrm{T_{SUM}} = \frac{\boldsymbol{J}^T\boldsymbol{\Sigma}^{-1}\boldsymbol{Z}}{\sqrt{\boldsymbol{J}^T\boldsymbol{\Sigma}^{-1}\boldsymbol{J}}} \sim \mathrm{N}(0,1)$$

a weighted sum of the components of $\boldsymbol{Z}$ with the weights equal to $\boldsymbol{J}^T\boldsymbol{\Sigma}^{-1}$. SUM is most powerful when the genetic effects are homogeneous (concordant in direction), and it loses power when the effects are heterogeneous.

VC (heterogeneous effects):

Treating the per-trait effects as random (mean 0, variance τ) yields the variance-component score statistic

$$\mathrm{T_{VC}} = \boldsymbol{Z}^T\boldsymbol{\Sigma}^{-1}\boldsymbol{\Sigma}^{-1}\boldsymbol{Z}$$

which, relative to the classical Wald statistic ($\boldsymbol{Z}^T\boldsymbol{\Sigma}^{-1}\boldsymbol{Z}$), contains one additional $\boldsymbol{\Sigma}^{-1}$. Under the null, $\mathrm{T_{VC}}$ follows a mixture of chi-square distributions, $\sum_{k=1}^{K}\lambda_k\chi^2_{1,k}$, where the weights λ_k are the eigenvalues of the matrix $\boldsymbol{\Sigma}^{-1}$. The p-value of $\mathrm{T_{VC}}$ is obtained by Davies' method. VC is robust to heterogeneous effects, including effects of opposite sign across traits.

mix-Fisher (robust combination):

The SUM and VC score statistics are asymptotically independent under the null. mix-Fisher combines their p-values (p_{SUM} and p_{VC}) by Fisher's method,

$$\mathrm{T_F} = -2\,[\ln p_{SUM} + \ln p_{VC}] \sim \chi^2_4$$

so it detects both homogeneous and heterogeneous effects and is robust across effect directions. Consistent with this, in our analysis the mix-Fisher result encompassed the SNPs detected by the SUM and VC tests.

S1.3. Genome-wide Efficient Mixed-Model Association (GEMMA)

GEMMA fits a linear mixed model (LMM) that controls for sample relatedness and population stratification through a genome-wide genetic relatedness matrix, and computes exact association test statistics efficiently (Zhou and Stephens, 2012). For a single trait, the univariate LMM is

$$y = \boldsymbol{W}\alpha + x\beta + u + \varepsilon$$

with random terms

$$u \sim N(0, \lambda\tau^{-1}\,\boldsymbol{K_{GRM}}), \quad \varepsilon \sim N(0, \tau^{-1}\,\boldsymbol{I_n})$$

where y is the n-vector of the trait, $\boldsymbol{W}$ the covariate matrix with coefficients alpha, x the genotype vector with effect β, K_{GRM} the centered genetic relatedness matrix, $\boldsymbol{I_n}$ the identity, τ^{-1} the residual variance, and λ the ratio of the genetic to residual variance components. Association is tested as H_0: $\beta = 0$.

In this study the six MetS-related traits were analyzed jointly using the multivariate extension of this model,

$$\boldsymbol{Y} = \boldsymbol{WA} + x\beta^T + \boldsymbol{G} + \boldsymbol{E}$$

$$vec(\boldsymbol{G}) \sim N(0, \boldsymbol{V}_g \otimes \boldsymbol{K_{GRM}}), \quad vec(E) \sim N(0, \boldsymbol{V_e} \otimes \boldsymbol{I_n})$$

where $\boldsymbol{Y}$ ($n \times 6$) stacks the six traits, $\boldsymbol{A}$ the covariate effects, β the six-vector of per-trait genotype effects, and $\boldsymbol{V_g}$ and $\boldsymbol{V_e}$ the 6 × 6 genetic and residual covariance matrices; $\boldsymbol{K_{GRM}}$ is the centered genetic relatedness matrix, $\boldsymbol{I_n}$ the identity, and $\otimes$ denotes the Kronecker product.

For each SNP, GEMMA evaluates the likelihood together with its first and second derivatives after a single eigen-decomposition of $\boldsymbol{K_{GRM}}$, which enables exact and computationally efficient calculation of the Wald, likelihood-ratio (LRT), and score statistics for H_0: $\beta = 0$ (each asymptotically chi-square, with degrees of freedom equal to the number of jointly modeled traits). The LRT showed genomic inflation in the discovery cohort (Figure S3) and was excluded from downstream analysis, and the Wald and score tests were retained.

Table S1. Association results for the 79 significant genetic variants identified by the multivariate analyses (MPAT and GEMMA) across the discovery and two replication cohorts

CHR	*SNP*	Study	MPAT			GEMMA	
			SUM	VC	mixFisher	WALD	SCORE
2	rs1260326	Discovery	6.14×10^{-01}	9.27×10^{-06}	4.69×10^{-05}	**5.28×10^{-13}**	**8.80×10^{-13}**
		Replication I	3.68×10^{-01}	**2.44×10^{-10}**	**3.84×10^{-10}**	**1.29×10^{-21}**	**3.61×10^{-21}**
		Replication II	3.17×10^{-02}	**3.68×10^{-62}**	**1.85×10^{-65}**	**4.44×10^{-158}**	**1.87×10^{-150}**
2	rs1260333	Discovery	3.51×10^{-01}	8.10×10^{-05}	2.87×10^{-04}	**2.32×10^{-09}**	**3.51×10^{-09}**
		Replication I	4.93×10^{-01}	**2.34×10^{-09}**	**5.75×10^{-09}**	**2.00×10^{-19}**	**5.08×10^{-19}**
		Replication II	2.20×10^{-02}	**4.16×10^{-51}**	**3.99×10^{-54}**	**1.92×10^{-113}**	**1.53×10^{-106}**
2	rs780092	Discovery	3.14×10^{-01}	7.48×10^{-05}	1.22×10^{-04}	**1.14×10^{-08}**	**1.68×10^{-08}**
		Replication I	6.97×10^{-01}	4.00×10^{-07}	1.60×10^{-06}	**4.42×10^{-13}**	**7.20×10^{-13}**
		Replication II	8.58×10^{-02}	**1.09×10^{-33}**	**4.26×10^{-35}**	**2.10×10^{-69}**	**4.40×10^{-67}**
2	rs780093	Discovery	4.12×10^{-01}	2.80×10^{-05}	1.01×10^{-04}	**2.13×10^{-10}**	**3.40×10^{-10}**
		Replication I	3.85×10^{-01}	**9.08×10^{-10}**	**1.63×10^{-09}**	**4.52×10^{-20}**	**1.18×10^{-19}**
		Replication II	1.38×10^{-02}	**8.15×10^{-52}**	**4.26×10^{-55}**	**7.34×10^{-117}**	**7.35×10^{-110}**
2	rs780094	Discovery	5.71×10^{-01}	2.45×10^{-05}	1.13×10^{-04}	**7.03×10^{-11}**	**1.19×10^{-10}**
		Replication I	3.31×10^{-01}	**2.02×10^{-09}**	**3.14×10^{-09}**	**2.98×10^{-19}**	**7.50×10^{-19}**
		Replication II	1.05×10^{-02}	**4.86×10^{-52}**	**1.77×10^{-55}**	**2.47×10^{-119}**	**2.45×10^{-112}**
8	rs10090948	Discovery	5.72×10^{-04}	5.64×10^{-04}	1.05×10^{-05}	**4.90×10^{-11}**	**5.41×10^{-09}**
		Replication I	1.94×10^{-02}	3.08×10^{-03}	9.94×10^{-04}	2.18×10^{-07}	3.03×10^{-07}
		Replication II	**1.20×10^{-18}**	**1.45×10^{-14}**	**1.47×10^{-26}**	**2.09×10^{-31}**	**3.77×10^{-30}**
8	rs10096633	Discovery	1.29×10^{-06}	9.27×10^{-06}	**7.02×10^{-10}**	**8.77×10^{-17}**	**8.11×10^{-15}**
		Replication I	6.05×10^{-04}	3.16×10^{-04}	8.01×10^{-06}	**9.01×10^{-13}**	**1.64×10^{-12}**
		Replication II	**2.19×10^{-33}**	**8.77×10^{-21}**	**6.33×10^{-44}**	**1.38×10^{-67}**	**4.79×10^{-65}**
8	rs10103634	Discovery	2.41×10^{-05}	2.19×10^{-04}	3.60×10^{-07}	**1.70×10^{-12}**	**1.49×10^{-10}**
		Replication I	1.72×10^{-02}	6.84×10^{-03}	1.66×10^{-03}	1.42×10^{-06}	1.84×10^{-06}
		Replication II	**9.90×10^{-19}**	**5.47×10^{-14}**	**3.58×10^{-26}**	**1.76×10^{-37}**	**1.60×10^{-36}**
8	rs10105606	Discovery	1.46×10^{-03}	1.44×10^{-04}	5.79×10^{-06}	**7.03×10^{-13}**	**3.37×10^{-11}**
		Replication I	1.77×10^{-03}	8.48×10^{-04}	5.47×10^{-05}	**9.42×10^{-11}**	**1.52×10^{-10}**
		Replication II	**3.06×10^{-27}**	**3.99×10^{-17}**	**1.79×10^{-35}**	**2.85×10^{-42}**	**3.27×10^{-40}**
8	rs10503669	Discovery	9.16×10^{-07}	9.27×10^{-06}	**5.40×10^{-10}**	**6.99×10^{-17}**	**5.94×10^{-15}**
		Replication I	9.64×10^{-04}	3.42×10^{-04}	1.22×10^{-05}	**1.28×10^{-12}**	**2.27×10^{-12}**
		Replication II	**2.28×10^{-33}**	**7.37×10^{-21}**	**5.25×10^{-44}**	**2.81×10^{-68}**	**9.89×10^{-66}**
8	rs11991231	Discovery	3.23×10^{-06}	6.25×10^{-05}	**1.71×10^{-08}**	**5.59×10^{-16}**	**2.33×10^{-13}**
		Replication I	3.02×10^{-03}	1.84×10^{-03}	1.35×10^{-04}	**3.56×10^{-09}**	**5.07×10^{-09}**
		Replication II	**6.24×10^{-24}**	**6.31×10^{-17}**	**4.00×10^{-33}**	**3.23×10^{-45}**	**1.57×10^{-43}**
8	rs12678919	Discovery	9.84×10^{-07}	9.32×10^{-06}	**4.95×10^{-10}**	**6.71×10^{-17}**	**5.62×10^{-15}**
		Replication I	8.70×10^{-04}	3.42×10^{-04}	1.13×10^{-05}	**1.28×10^{-12}**	**2.30×10^{-12}**
		Replication II	**3.09×10^{-33}**	**8.10×10^{-21}**	**7.31×10^{-44}**	**7.89×10^{-68}**	**2.73×10^{-65}**
8	rs13702	Discovery	5.47×10^{-04}	1.65×10^{-04}	2.79×10^{-06}	**9.90×10^{-13}**	**4.72×10^{-11}**
		Replication I	9.42×10^{-04}	8.67×10^{-04}	3.62×10^{-05}	**7.60×10^{-11}**	**1.21×10^{-10}**
		Replication II	**2.99×10^{-27}**	**2.25×10^{-17}**	**9.09×10^{-36}**	**2.75×10^{-43}**	**3.68×10^{-41}**
8	rs15285	Discovery	5.97×10^{-04}	1.66×10^{-04}	3.02×10^{-06}	**8.38×10^{-13}**	**4.24×10^{-11}**
		Replication I	7.48×10^{-04}	7.81×10^{-13}	2.80×10^{-05}	**5.26×10^{-11}**	**8.41×10^{-11}**
		Replication II	**4.00×10^{-27}**	**2.10×10^{-17}**	**1.04×10^{-35}**	**2.21×10^{-43}**	**3.01×10^{-41}**
8	rs1534650	Discovery	3.49×10^{-04}	2.47×10^{-04}	2.79×10^{-06}	**3.19×10^{-13}**	**5.60×10^{-11}**
		Replication I	3.18×10^{-03}	1.30×10^{-03}	1.26×10^{-04}	**2.92×10^{-09}**	**4.65×10^{-09}**
		Replication II	**1.43×10^{-19}**	**2.03×10^{-14}**	**4.80×10^{-27}**	**2.96×10^{-28}**	**5.22×10^{-27}**
8	rs17091905	Discovery	1.05×10^{-06}	9.29×10^{-06}	**5.48×10^{-10}**	**6.34×10^{-17}**	**5.76×10^{-15}**
		Replication I	1.02×10^{-03}	3.17×10^{-04}	1.18×10^{-05}	**1.07×10^{-12}**	**1.92×10^{-12}**
		Replication II	**2.52×10^{-33}**	**9.19×10^{-21}**	**7.97×10^{-44}**	**1.69×10^{-67}**	**5.90×10^{-65}**
8	rs17411031	Discovery	4.81×10^{-04}	1.33×10^{-04}	1.96×10^{-06}	**2.71×10^{-13}**	**1.50×10^{-11}**
		Replication I	2.48×10^{-03}	6.56×10^{-04}	5.86×10^{-05}	**9.06×10^{-11}**	**1.50×10^{-10}**
		Replication II	**2.74×10^{-25}**	**7.91×10^{-17}**	**9.71×10^{-34}**	**3.21×10^{-39}**	**3.19×10^{-37}**
8	rs17482753	Discovery	1.29×10^{-06}	9.27×10^{-06}	**7.02×10^{-10}**	**8.77×10^{-17}**	**8.11×10^{-15}**
		Replication I	5.50×10^{-04}	3.11×10^{-04}	7.36×10^{-06}	**7.85×10^{-13}**	**1.44×10^{-12}**
		Replication II	**4.12×10^{-33}**	**7.47×10^{-21}**	**8.63×10^{-44}**	**7.18×10^{-68}**	**2.47×10^{-65}**
8	rs2083637	Discovery	6.02×10^{-04}	1.47×10^{-04}	2.70×10^{-06}	**4.50×10^{-13}**	**2.50×10^{-11}**
		Replication I	9.57×10^{-04}	4.55×10^{-04}	2.10×10^{-05}	**1.70×10^{-11}**	**2.92×10^{-11}**
		Replication II	**1.98×10^{-25}**	**8.07×10^{-17}**	**7.49×10^{-34}**	**1.27×10^{-39}**	**1.26×10^{-37}**
8	rs2197089	Discovery	5.28×10^{-04}	1.83×10^{-03}	2.94×10^{-05}	**5.97×10^{-10}**	**2.38×10^{-08}**
		Replication I	9.52×10^{-04}	4.37×10^{-03}	1.73×10^{-04}	3.22×10^{-07}	4.69×10^{-07}
		Replication II	**2.30×10^{-18}**	**4.03×10^{-15}**	**1.09×10^{-26}**	**1.35×10^{-29}**	**2.37×10^{-28}**
8	rs271	Discovery	3.49×10^{-03}	1.62×10^{-03}	1.14×10^{-04}	**1.55×10^{-08}**	4.46×10^{-07}
		Replication I	1.46×10^{-03}	1.04×10^{-02}	5.90×10^{-04}	7.88×10^{-06}	1.01×10^{-05}
		Replication II	**5.89×10^{-12}**	**1.58×10^{-09}**	**8.85×10^{-17}**	**2.61×10^{-14}**	**8.52×10^{-14}**
8	rs28526159	Discovery	3.39×10^{-04}	5.06×10^{-04}	6.01×10^{-06}	**2.14×10^{-11}**	**2.20×10^{-09}**
		Replication I	2.89×10^{-02}	3.68×10^{-03}	1.54×10^{-03}	6.91×10^{-07}	9.49×10^{-07}
		Replication II	**5.50×10^{-20}**	**1.15×10^{-14}**	**1.15×10^{-27}**	**1.26×10^{-32}**	**2.38×10^{-31}**
8	rs295	Discovery	6.48×10^{-04}	1.78×10^{-04}	3.43×10^{-06}	**2.72×10^{-12}**	**1.87×10^{-10}**

		Replication I	9.18×10^{-05}	3.57×10^{-04}	2.98×10^{-06}	**1.16×10^{-12}**	**2.06×10^{-12}**
		Replication II	**4.86×10^{-27}**	**1.26×10^{-17}**	**6.14×10^{-36}**	**6.33×10^{-45}**	**8.34×10^{-43}**
8	rs301	Discovery	5.19×10^{-03}	9.42×10^{-04}	9.31×10^{-05}	**8.51×10^{-10}**	**3.02×10^{-08}**
		Replication I	1.37×10^{-02}	4.15×10^{-03}	1.09×10^{-03}	**1.72×10^{-08}**	**2.50×10^{-08}**
		Replication II	**2.06×10^{-21}**	**2.97×10^{-12}**	**3.03×10^{-26}**	**1.34×10^{-20}**	**1.18×10^{-19}**
8	rs320	Discovery	1.03×10^{-03}	1.87×10^{-04}	5.39×10^{-06}	**3.43×10^{-12}**	**1.40×10^{-10}**
		Replication I	7.72×10^{-04}	1.09×10^{-03}	3.93×10^{-05}	**1.50×10^{-10}**	**2.39×10^{-10}**
		Replication II	**1.99×10^{-26}**	**4.65×10^{-17}**	**7.14×10^{-35}**	**1.40×10^{-41}**	**1.55×10^{-39}**
8	rs325	Discovery	9.09×10^{-07}	1.11×10^{-05}	**1.08×10^{-09}**	**5.76×10^{-16}**	**4.36×10^{-14}**
		Replication I	8.78×10^{-04}	3.13×10^{-04}	1.07×10^{-05}	**6.14×10^{-13}**	**1.09×10^{-12}**
		Replication II	**2.21×10^{-33}**	**7.66×10^{-21}**	**6.00×10^{-44}**	**5.56×10^{-68}**	**1.84×10^{-65}**
8	rs326	Discovery	7.55×10^{-04}	1.72×10^{-04}	3.86×10^{-06}	**1.48×10^{-12}**	**6.39×10^{-11}**
		Replication I	7.87×10^{-04}	9.67×10^{-04}	3.56×10^{-05}	**1.12×10^{-10}**	**1.80×10^{-10}**
		Replication II	**4.73×10^{-27}**	**2.51×10^{-17}**	**1.38×10^{-35}**	**3.82×10^{-43}**	**4.70×10^{-41}**
8	rs328	Discovery	9.99×10^{-07}	1.09×10^{-05}	**1.01×10^{-09}**	**4.35×10^{-16}**	**3.26×10^{-14}**
		Replication I	7.83×10^{-04}	2.94×10^{-04}	9.17×10^{-06}	**4.68×10^{-13}**	**8.44×10^{-13}**
		Replication II	**1.74×10^{-33}**	**7.31×10^{-21}**	**4.64×10^{-44}**	**3.68×10^{-68}**	**1.28×10^{-65}**
8	rs4244457	Discovery	2.11×10^{-04}	9.18×10^{-04}	6.83×10^{-06}	**5.83×10^{-11}**	**5.84×10^{-09}**
		Replication I	5.93×10^{-03}	4.31×10^{-03}	5.26×10^{-04}	1.90×10^{-07}	2.63×10^{-07}
		Replication II	**1.69×10^{-21}**	**9.18×10^{-16}**	**7.02×10^{-30}**	**2.97×10^{-38}**	**8.23×10^{-37}**
8	rs4490856	Discovery	6.25×10^{-04}	6.31×10^{-04}	1.27×10^{-05}	**9.57×10^{-12}**	**6.36×10^{-10}**
		Replication I	4.22×10^{-04}	1.46×10^{-03}	2.94×10^{-05}	**4.42×10^{-09}**	**7.41×10^{-09}**
		Replication II	**5.36×10^{-20}**	**1.98×10^{-15}**	**2.49×10^{-28}**	**2.80×10^{-32}**	**7.22×10^{-31}**
8	rs7016880	Discovery	1.44×10^{-06}	1.13×10^{-05}	**1.61×10^{-09}**	**6.90×10^{-17}**	**1.04×10^{-14}**
		Replication I	6.17×10^{-04}	4.13×10^{-04}	1.07×10^{-05}	**2.43×10^{-12}**	**4.03×10^{-12}**
		Replication II	**1.05×10^{-30}**	**2.20×10^{-19}**	**1.52×10^{-40}**	**8.30×10^{-59}**	**1.55×10^{-56}**
8	rs7816032	Discovery	9.82×10^{-03}	2.62×10^{-04}	5.14×10^{-05}	**2.97×10^{-10}**	**1.32×10^{-08}**
		Replication I	4.60×10^{-05}	1.29×10^{-03}	6.90×10^{-06}	2.50×10^{-07}	3.65×10^{-07}
		Replication II	**2.52×10^{-13}**	**6.44×10^{-09}**	**4.53×10^{-17}**	**2.07×10^{-13}**	**5.11×10^{-13}**
8	rs7841189	Discovery	9.00×10^{-07}	9.15×10^{-06}	**4.51×10^{-10}**	**4.20×10^{-17}**	**3.72×10^{-15}**
		Replication I	1.73×10^{-03}	3.23×10^{-04}	1.84×10^{-05}	**1.26×10^{-12}**	**2.20×10^{-12}**
		Replication II	**2.74×10^{-32}**	**7.50×10^{-20}**	**3.34×10^{-42}**	**5.47×10^{-62}**	**1.41×10^{-59}**
8	rs894210	Discovery	3.41×10^{-04}	5.96×10^{-04}	7.38×10^{-06}	**4.49×10^{-12}**	**2.89×10^{-10}**
		Replication I	3.42×10^{-04}	1.45×10^{-03}	2.72×10^{-05}	**5.01×10^{-09}**	**8.59×10^{-09}**
		Replication II	**1.06×10^{-19}**	**5.09×10^{-15}**	**1.07×10^{-27}**	**7.54×10^{-31}**	**1.68×10^{-29}**
9	rs1883025	Discovery	1.73×10^{-01}	3.17×10^{-04}	6.62×10^{-04}	**1.25×10^{-08}**	**2.51×10^{-08}**
		Replication I	4.39×10^{-04}	7.17×10^{-04}	2.86×10^{-05}	2.21×10^{-06}	2.40×10^{-06}
		Replication II	1.46×10^{-06}	**1.48×10^{-35}**	**9.66×10^{-38}**	**2.23×10^{-85}**	**2.77×10^{-84}**
9	rs2575876	Discovery	1.55×10^{-01}	4.89×10^{-04}	9.29×10^{-04}	**7.03×10^{-08}**	**1.27×10^{-07}**
		Replication I	3.62×10^{-04}	6.14×10^{-04}	2.17×10^{-05}	1.51×10^{-06}	1.65×10^{-06}
		Replication II	2.07×10^{-07}	**1.32×10^{-35}**	**2.69×10^{-38}**	**2.05×10^{-86}**	**2.48×10^{-85}**
11	rs10790162	Discovery	1.09×10^{-05}	1.37×10^{-04}	**4.13×10^{-08}**	**8.02×10^{-14}**	**1.09×10^{-12}**
		Replication I	2.30×10^{-06}	1.60×10^{-07}	**2.71×10^{-11}**	**8.13×10^{-23}**	**3.07×10^{-22}**
		Replication II	**6.20×10^{-32}**	**9.44×10^{-35}**	**9.14×10^{-60}**	**3.62×10^{-112}**	**1.46×10^{-104}**
11	rs11216126	Discovery	2.49×10^{-04}	7.66×10^{-03}	4.53×10^{-05}	**2.78×10^{-09}**	**6.86×10^{-09}**
		Replication I	4.02×10^{-05}	5.27×10^{-06}	**3.72×10^{-08}**	**6.88×10^{-17}**	**1.28×10^{-16}**
		Replication II	**6.39×10^{-27}**	**3.45×10^{-25}**	**1.71×10^{-44}**	**1.16×10^{-118}**	**4.24×10^{-116}**
11	rs11602073	Discovery	8.06×10^{-05}	4.55×10^{-03}	1.09×10^{-05}	**1.45×10^{-10}**	**5.02×10^{-10}**
		Replication I	2.45×10^{-06}	1.63×10^{-07}	**7.75×10^{-11}**	**1.25×10^{-20}**	**2.81×10^{-20}**
		Replication II	**8.66×10^{-30}**	**1.92×10^{-27}**	**5.08×10^{-49}**	**7.89×10^{-131}**	**9.59×10^{-128}**
11	rs1558861	Discovery	8.97×10^{-06}	2.32×10^{-04}	**5.71×10^{-08}**	**7.10×10^{-13}**	**8.97×10^{-12}**
		Replication I	3.75×10^{-05}	2.43×10^{-07}	**4.00×10^{-10}**	**3.82×10^{-22}**	**1.31×10^{-21}**
		Replication II	**3.90×10^{-30}**	**6.98×10^{-34}**	**1.65×10^{-57}**	**6.05×10^{-106}**	**1.39×10^{-98}**
11	rs2075290	Discovery	9.97×10^{-06}	2.54×10^{-04}	**7.09×10^{-08}**	**5.45×10^{-13}**	**8.57×10^{-12}**
		Replication I	1.33×10^{-06}	**3.20×10^{-08}**	**3.16×10^{-12}**	**4.40×10^{-25}**	**2.04×10^{-24}**
		Replication II	**2.75×10^{-31}**	**3.49×10^{-34}**	**9.80×10^{-59}**	**1.28×10^{-108}**	**4.98×10^{-101}**
11	rs2075291	Discovery	**2.87×10^{-14}**	**9.28×10^{-09}**	**3.36×10^{-19}**	**8.12×10^{-42}**	**1.97×10^{-39}**
		Replication I	**1.19×10^{-09}**	**2.12×10^{-12}**	**6.29×10^{-19}**	**5.26×10^{-44}**	**9.12×10^{-43}**
		Replication II	**1.69×10^{-77}**	**4.01×10^{-59}**	**4.52×10^{-117}**	**$0.00\times10^{+00}$**	**$0.00\times10^{+00}$**
11	rs2075295	Discovery	**6.42×10^{-08}**	4.18×10^{-04}	**3.80×10^{-09}**	**5.24×10^{-12}**	**8.73×10^{-12}**
		Replication I	7.08×10^{-05}	1.13×10^{-05}	**2.23×10^{-08}**	**3.65×10^{-22}**	**8.64×10^{-22}**
		Replication II	**3.83×10^{-39}**	**1.89×10^{-24}**	**4.40×10^{-53}**	**3.42×10^{-122}**	**2.29×10^{-119}**
11	rs2266788	Discovery	1.70×10^{-05}	1.44×10^{-04}	**6.17×10^{-08}**	**9.29×10^{-14}**	**1.42×10^{-12}**
		Replication I	4.45×10^{-07}	**2.50×10^{-08}**	**9.60×10^{-13}**	**8.93×10^{-26}**	**4.08×10^{-25}**
		Replication II	**1.31×10^{-32}**	**4.04×10^{-36}**	**7.84×10^{-62}**	**3.86×10^{-118}**	**3.19×10^{-110}**
11	rs5128	Discovery	4.72×10^{-01}	6.24×10^{-04}	1.94×10^{-03}	**1.27×10^{-07}**	5.04×10^{-07}
		Replication I	1.65×10^{-01}	5.21×10^{-04}	5.32×10^{-04}	6.90×10^{-07}	8.18×10^{-07}
		Replication II	**9.40×10^{-12}**	**1.05×10^{-16}**	**5.27×10^{-25}**	**7.62×10^{-35}**	**1.23×10^{-33}**
11	rs603446	Discovery	1.01×10^{-02}	1.15×10^{-04}	1.27×10^{-05}	**1.94×10^{-13}**	**1.95×10^{-12}**
		Replication I	2.27×10^{-04}	1.02×10^{-06}	**4.02×10^{-09}**	**7.33×10^{-16}**	**1.31×10^{-15}**
		Replication II	**5.71×10^{-14}**	**1.13×10^{-24}**	**6.75×10^{-35}**	**6.28×10^{-93}**	**1.56×10^{-91}**
11	rs6589566	Discovery	1.09×10^{-05}	1.26×10^{-04}	**3.60×10^{-08}**	**3.94×10^{-14}**	**6.40×10^{-13}**
		Replication I	6.61×10^{-07}	**2.52×10^{-08}**	**1.37×10^{-12}**	**1.41×10^{-25}**	**6.59×10^{-25}**
		Replication II	**2.64×10^{-32}**	**4.93×10^{-36}**	**1.75×10^{-61}**	**2.24×10^{-117}**	**1.84×10^{-109}**

11	rs662799	Discovery	**7.97×10^{-17}**	**3.13×10^{-12}**	**1.98×10^{-25}**	**2.70×10^{-53}**	**4.24×10^{-49}**
		Replication I	**3.80×10^{-16}**	**2.82×10^{-21}**	**1.61×10^{-33}**	**1.60×10^{-76}**	**7.58×10^{-73}**
		Replication II	**1.70×10^{-105}**	**1.54×10^{-96}**	**2.44×10^{-180}**	**$0.00\times10^{+00}$**	**$0.00\times10^{+00}$**
11	rs964184	Discovery	1.35×10^{-05}	1.42×10^{-04}	**5.18×10^{-08}**	**6.15×10^{-14}**	**1.18×10^{-12}**
		Replication I	1.05×10^{-06}	**1.10×10^{-08}**	**9.65×10^{-13}**	**1.77×10^{-26}**	**8.63×10^{-26}**
		Replication II	**7.23×10^{-32}**	**8.64×10^{-36}**	**7.24×10^{-61}**	**1.52×10^{-116}**	**9.04×10^{-109}**
12	rs11066280	Discovery	7.39×10^{-03}	2.84×10^{-07}	**1.40×10^{-08}**	**1.93×10^{-20}**	**2.78×10^{-19}**
		Replication I	3.29×10^{-05}	4.16×10^{-05}	**3.96×10^{-08}**	**2.34×10^{-13}**	**4.26×10^{-13}**
		Replication II	1.75×10^{-07}	**7.55×10^{-22}**	**1.71×10^{-26}**	**1.89×10^{-66}**	**8.46×10^{-65}**
12	rs11066359	Discovery	3.54×10^{-02}	1.39×10^{-03}	3.07×10^{-04}	**4.42×10^{-08}**	**1.10×10^{-07}**
		Replication I	5.89×10^{-04}	1.25×10^{-02}	1.63×10^{-04}	1.99×10^{-05}	2.25×10^{-05}
		Replication II	1.03×10^{-04}	**1.67×10^{-08}**	**9.47×10^{-11}**	**3.31×10^{-17}**	**7.81×10^{-17}**
12	rs11066453	Discovery	1.81×10^{-01}	7.62×10^{-04}	8.16×10^{-04}	**1.84×10^{-08}**	**8.67×10^{-08}**
		Replication I	3.35×10^{-04}	3.69×10^{-02}	3.04×10^{-04}	5.02×10^{-05}	5.86×10^{-05}
		Replication II	1.71×10^{-02}	**1.10×10^{-09}**	**3.14×10^{-10}**	**8.98×10^{-17}**	**2.08×10^{-16}**
12	rs12229654	Discovery	3.43×10^{-02}	2.69×10^{-05}	7.47×10^{-06}	**7.93×10^{-14}**	**3.49×10^{-13}**
		Replication I	2.85×10^{-04}	1.93×10^{-03}	1.49×10^{-05}	**6.43×10^{-08}**	**7.78×10^{-08}**
		Replication II	2.03×10^{-06}	**4.20×10^{-14}**	**8.50×10^{-18}**	**8.50×10^{-40}**	**8.64×10^{-39}**
12	rs2072134	Discovery	7.06×10^{-02}	3.54×10^{-04}	1.57×10^{-04}	**5.28×10^{-10}**	**2.93×10^{-09}**
		Replication I	8.30×10^{-05}	1.11×10^{-02}	2.79×10^{-05}	1.78×10^{-06}	2.22×10^{-06}
		Replication II	7.94×10^{-03}	**3.60×10^{-10}**	**5.60×10^{-11}**	**1.55×10^{-19}**	**4.26×10^{-19}**
12	rs2074356	Discovery	5.21×10^{-03}	6.45×10^{-06}	5.35×10^{-07}	**4.59×10^{-18}**	**6.70×10^{-17}**
		Replication I	1.76×10^{-05}	7.23×10^{-06}	**6.29×10^{-09}**	**2.45×10^{-15}**	**4.46×10^{-15}**
		Replication II	**1.03×10^{-07}**	**8.68×10^{-23}**	**1.37×10^{-27}**	**2.92×10^{-71}**	**1.58×10^{-69}**
12	rs671	Discovery	2.88×10^{-03}	2.82×10^{-07}	**5.58×10^{-09}**	**7.98×10^{-21}**	**1.13×10^{-19}**
		Replication I	3.33×10^{-06}	7.32×10^{-06}	**1.38×10^{-09}**	**5.93×10^{-16}**	**1.14×10^{-15}**
		Replication II	**2.62×10^{-08}**	**1.40×10^{-24}**	**5.15×10^{-30}**	**4.93×10^{-81}**	**2.98×10^{-79}**
12	rs77768175	Discovery	2.19×10^{-03}	2.48×10^{-07}	**3.70×10^{-09}**	**2.15×10^{-21}**	**3.48×10^{-20}**
		Replication I	6.93×10^{-06}	1.77×10^{-05}	**4.23×10^{-09}**	**5.74×10^{-15}**	**1.07×10^{-14}**
		Replication II	**2.59×10^{-08}**	**1.53×10^{-23}**	**5.90×10^{-29}**	**9.42×10^{-77}**	**5.10×10^{-75}**
15	rs10468017	Discovery	2.53×10^{-02}	3.11×10^{-06}	2.60×10^{-06}	**4.59×10^{-12}**	**2.45×10^{-11}**
		Replication I	7.26×10^{-04}	**1.18×10^{-08}**	**1.69×10^{-09}**	**9.97×10^{-16}**	**1.67×10^{-15}**
		Replication II	1.54×10^{-05}	**4.00×10^{-45}**	**1.09×10^{-46}**	**4.20×10^{-95}**	**3.98×10^{-93}**
15	rs11635491	Discovery	1.21×10^{-01}	3.74×10^{-07}	2.65×10^{-06}	**4.94×10^{-14}**	**1.55×10^{-13}**
		Replication I	1.02×10^{-02}	**1.29×10^{-15}**	**1.26×10^{-15}**	**1.56×10^{-29}**	**1.18×10^{-28}**
		Replication II	**5.81×10^{-12}**	**2.38×10^{-57}**	**1.30×10^{-62}**	**6.89×10^{-126}**	**1.14×10^{-121}**
15	rs1532085	Discovery	3.31×10^{-04}	**5.94×10^{-11}**	**3.29×10^{-12}**	**1.06×10^{-20}**	**4.17×10^{-19}**
		Replication I	6.25×10^{-04}	**2.03×10^{-12}**	**3.90×10^{-13}**	**3.01×10^{-23}**	**8.93×10^{-23}**
		Replication II	**4.92×10^{-13}**	**2.60×10^{-58}**	**3.04×10^{-64}**	**2.39×10^{-132}**	**2.72×10^{-128}**
15	rs16940212	Discovery	1.26×10^{-03}	2.44×10^{-05}	1.85×10^{-06}	**9.83×10^{-11}**	**7.86×10^{-10}**
		Replication I	3.56×10^{-01}	8.29×10^{-07}	3.92×10^{-06}	**8.88×10^{-11}**	**1.21×10^{-10}**
		Replication II	4.68×10^{-04}	**1.68×10^{-31}**	**3.27×10^{-32}**	**4.80×10^{-55}**	**1.28×10^{-53}**
15	rs1800588	Discovery	6.41×10^{-03}	**4.81×10^{-13}**	**2.38×10^{-13}**	**1.57×10^{-30}**	**3.48×10^{-29}**
		Replication I	2.25×10^{-04}	**9.74×10^{-24}**	**1.39×10^{-24}**	**1.68×10^{-48}**	**6.83×10^{-47}**
		Replication II	**3.68×10^{-17}**	**8.00×10^{-98}**	**5.35×10^{-106}**	**9.13×10^{-242}**	**5.36×10^{-233}**
15	rs2043085	Discovery	2.01×10^{-04}	**9.00×10^{-10}**	**3.02×10^{-11}**	**6.77×10^{-19}**	**2.05×10^{-17}**
		Replication I	8.49×10^{-04}	**9.30×10^{-12}**	**2.01×10^{-12}**	**1.68×10^{-21}**	**4.56×10^{-21}**
		Replication II	**2.42×10^{-11}**	**9.34×10^{-53}**	**1.07×10^{-57}**	**9.76×10^{-116}**	**3.21×10^{-112}**
15	rs261334	Discovery	2.48×10^{-02}	**1.73×10^{-10}**	**1.94×10^{-10}**	**2.68×10^{-23}**	**2.87×10^{-22}**
		Replication I	1.40×10^{-03}	**1.63×10^{-22}**	**6.10×10^{-23}**	**7.76×10^{-45}**	**2.26×10^{-43}**
		Replication II	**1.12×10^{-13}**	**4.59×10^{-83}**	**2.81×10^{-89}**	**1.48×10^{-202}**	**8.61×10^{-196}**
15	rs28528324	Discovery	3.10×10^{-03}	1.06×10^{-04}	1.32×10^{-05}	**1.55×10^{-09}**	**6.21×10^{-09}**
		Replication I	1.95×10^{-01}	9.18×10^{-07}	3.14×10^{-06}	**9.89×10^{-11}**	**1.33×10^{-10}**
		Replication II	6.31×10^{-03}	**2.44×10^{-25}**	**1.87×10^{-25}**	**1.68×10^{-40}**	**1.52×10^{-39}**
15	rs4775041	Discovery	2.59×10^{-02}	4.97×10^{-06}	3.33×10^{-06}	**7.46×10^{-12}**	**3.87×10^{-11}**
		Replication I	8.71×10^{-04}	**1.10×10^{-08}**	**1.76×10^{-09}**	**6.27×10^{-16}**	**1.06×10^{-15}**
		Replication II	2.47×10^{-05}	**2.37×10^{-45}**	**8.32×10^{-47}**	**3.11×10^{-95}**	**3.11×10^{-93}**
15	rs79341002	Discovery	2.05×10^{-02}	9.19×10^{-06}	3.45×10^{-06}	**2.93×10^{-12}**	**1.26×10^{-11}**
		Replication I	1.49×10^{-03}	**2.85×10^{-09}**	**7.14×10^{-10}**	**1.48×10^{-16}**	**2.73×10^{-16}**
		Replication II	1.69×10^{-07}	**5.21×10^{-45}**	**1.19×10^{-47}**	**6.14×10^{-97}**	**7.79×10^{-95}**
16	rs1532624	Discovery	3.91×10^{-05}	9.50×10^{-06}	**3.51×10^{-08}**	**2.31×10^{-17}**	**2.85×10^{-16}**
		Replication I	1.28×10^{-04}	3.38×10^{-07}	**3.93×10^{-08}**	**1.18×10^{-14}**	**2.41×10^{-14}**
		Replication II	**3.33×10^{-34}**	**3.01×10^{-58}**	**2.90×10^{-78}**	**1.64×10^{-144}**	**5.64×10^{-137}**
16	rs173539	Discovery	2.11×10^{-03}	1.68×10^{-04}	1.55×10^{-05}	**6.79×10^{-11}**	**4.03×10^{-10}**
		Replication I	2.20×10^{-05}	8.82×10^{-06}	**5.08×10^{-08}**	**7.91×10^{-13}**	**1.32×10^{-12}**
		Replication II	**3.50×10^{-19}**	**3.77×10^{-41}**	**2.04×10^{-51}**	**1.42×10^{-83}**	**1.09×10^{-79}**
16	rs247616	Discovery	1.60×10^{-07}	**1.49×10^{-10}**	**2.43×10^{-14}**	**4.11×10^{-32}**	**4.44×10^{-30}**
		Replication I	**2.09×10^{-09}**	**3.99×10^{-14}**	**1.85×10^{-18}**	**1.01×10^{-35}**	**1.50×10^{-34}**
		Replication II	**3.58×10^{-46}**	**1.81×10^{-109}**	**1.31×10^{-136}**	**5.51×10^{-313}**	**9.49×10^{-295}**
16	rs247617	Discovery	2.83×10^{-07}	**2.71×10^{-10}**	**6.59×10^{-14}**	**1.98×10^{-31}**	**2.46×10^{-29}**
		Replication I	**8.96×10^{-10}**	**2.05×10^{-14}**	**5.54×10^{-19}**	**2.73×10^{-36}**	**4.20×10^{-35}**
		Replication II	**1.67×10^{-47}**	**2.99×10^{-110}**	**3.23×10^{-138}**	**5.89×10^{-314}**	**1.25×10^{-295}**
16	rs3764261	Discovery	3.27×10^{-07}	**1.85×10^{-10}**	**5.19×10^{-14}**	**8.16×10^{-32}**	**9.33×10^{-30}**
		Replication I	**1.06×10^{-09}**	**1.72×10^{-14}**	**5.26×10^{-19}**	**1.31×10^{-36}**	**2.03×10^{-35}**

		Replication II	**1.45×10^{-47}**	**3.55×10^{-110}**	**3.37×10^{-138}**	**5.91×10^{-315}**	**1.48×10^{-296}**
		Discovery	1.07×10^{-04}	9.04×10^{-06}	**4.26×10^{-08}**	**4.17×10^{-17}**	**1.18×10^{-15}**
16	rs4783961	Replication I	**3.29×10^{-09}**	**8.64×10^{-09}**	**2.83×10^{-13}**	**2.86×10^{-21}**	**7.95×10^{-21}**
		Replication II	**2.30×10^{-29}**	**5.59×10^{-70}**	**1.92×10^{-86}**	**3.66×10^{-178}**	**1.57×10^{-169}**
		Discovery	1.02×10^{-05}	9.35×10^{-06}	**1.26×10^{-08}**	**2.77×10^{-16}**	**2.16×10^{-15}**
16	rs708272	Replication I	2.51×10^{-06}	**1.57×10^{-08}**	**5.12×10^{-11}**	**5.94×10^{-18}**	**1.40×10^{-17}**
		Replication II	**3.01×10^{-25}**	**1.07×10^{-47}**	**8.74×10^{-62}**	**6.16×10^{-111}**	**3.49×10^{-106}**
		Discovery	**1.28×10^{-07}**	2.42×10^{-07}	**4.37×10^{-11}**	**2.44×10^{-22}**	**9.10×10^{-21}**
16	rs72786786	Replication I	2.18×10^{-06}	**6.53×10^{-11}**	**2.59×10^{-13}**	**5.02×10^{-24}**	**2.01×10^{-23}**
		Replication II	**7.26×10^{-39}**	**3.40×10^{-82}**	**7.06×10^{-105}**	**8.19×10^{-213}**	**5.44×10^{-200}**
		Discovery	3.18×10^{-06}	3.13×10^{-05}	**2.00×10^{-08}**	**3.78×10^{-15}**	**5.62×10^{-14}**
16	rs7499892	Replication I	6.73×10^{-04}	1.18×10^{-04}	6.91×10^{-06}	**4.12×10^{-10}**	**5.17×10^{-10}**
		Replication II	**7.00×10^{-24}**	**2.74×10^{-42}**	**9.81×10^{-56}**	**1.30×10^{-130}**	**4.54×10^{-128}**
		Discovery	5.67×10^{-04}	6.01×10^{-04}	1.75×10^{-05}	**6.77×10^{-08}**	6.54×10^{-07}
16	rs9939224	Replication I	5.94×10^{-04}	2.62×10^{-03}	1.13×10^{-04}	2.24×10^{-06}	2.45×10^{-06}
		Replication II	**2.10×10^{-19}**	**3.47×10^{-27}**	**5.82×10^{-38}**	**6.27×10^{-74}**	**1.35×10^{-72}**
		Discovery	1.04×10^{-03}	4.32×10^{-03}	1.29×10^{-04}	**2.43×10^{-09}**	**1.58×10^{-09}**
19	rs429358	Replication I	5.49×10^{-01}	6.56×10^{-04}	2.84×10^{-03}	**6.66×10^{-10}**	**8.07×10^{-10}**
		Replication II	3.83×10^{-10}	3.06×10^{-12}	4.63×10^{-18}	3.56×10^{-56}	**1.04×10^{-55}**
		Discovery	5.65×10^{-03}	1.66×10^{-02}	1.55×10^{-03}	**1.16×10^{-07}**	**1.45×10^{-07}**
19	rs4420638	Replication I	7.56×10^{-01}	5.49×10^{-03}	2.38×10^{-02}	7.86×10^{-07}	8.82×10^{-07}
		Replication II	6.82×10^{-07}	**4.23×10^{-10}**	**1.34×10^{-13}**	**9.27×10^{-42}**	**2.10×10^{-41}**

P-values of 79 SNPs significantly associated with metabolic syndrome (MetS) are summarized across discovery and replication cohorts using Multiple Phenotype Association Tests (MPAT) (SUM, VC, mixFisher) and Genome-wide Efficient Mixed Model Association (GEMMA) (WALD, SCORE). Values satisfying the Bonferroni correction threshold (Replication I: $p<1.564\times10^{-7}$; Replication II: $p<1.566\times10^{-7}$) are shown in bold.

Table S2. Summary of the RegulomeDB scoring categories and their descriptions

RegulomeDB Category	Category Description
Likely to affect binding and linked to expression of a gene target	
1a	eQTL [a] + TF [b] binding + matched TF motif + matched DNase [c] footprint + DNase peak
1b	eQTL + TF binding + any motif + DNase footprint + DNase peak
1c	eQTL + TF binding + matched TF motif + DNase peak
1d	eQTL + TF binding + any motif + DNase peak
1e	eQTL + TF binding + matched TF motif
1f	eQTL + TF binding/DNase peak
Likely to affect binding	
2a	TF binding + matched TF motif + matched DNase footprint + DNase peak
2b	TF binding + any motif + DNase footprint + DNase peak
2c	TF binding + matched TF motif + DNase peak
Less likely to affect binding	
3a	TF binding + any motif + DNase peak
3b	TF binding + matched TF motif
Minimal binding evidence	
4	TF binding + DNase peak
5	TF binding or DNase peak
6	Motif hit

[a] Expression quantitative trait locus (eQTL); [b] transcription factor (TF); [c] deoxyribonuclease (DNase).

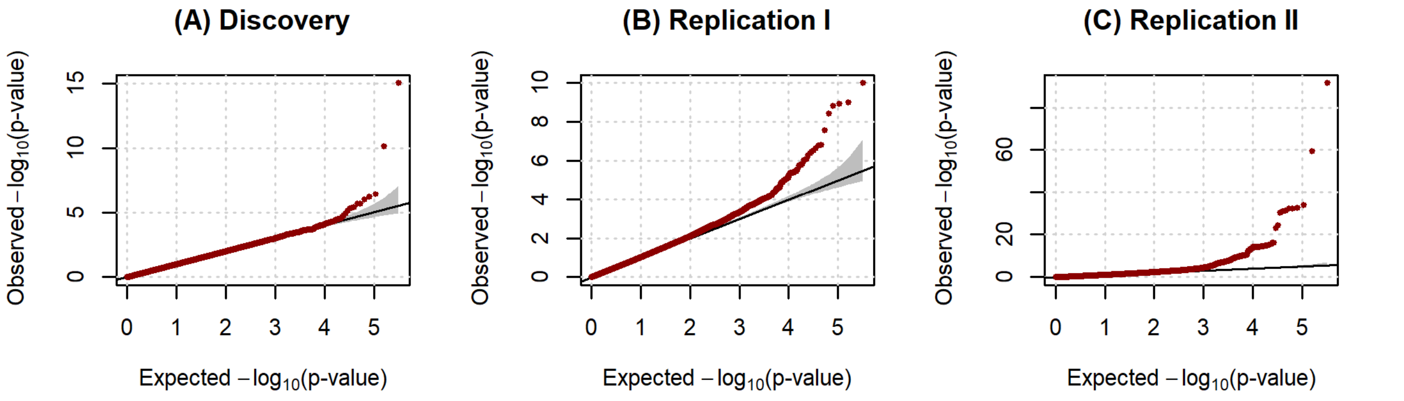

Figure S1. Quantile–quantile plots for the logistic regression analysis in the discovery and two replication cohorts. Red dots represent the observed $-\log_{10}$(P-values), and the black line denotes values expected under the null hypothesis of a uniform P-value distribution. Grey shading indicates the 95% confidence interval.

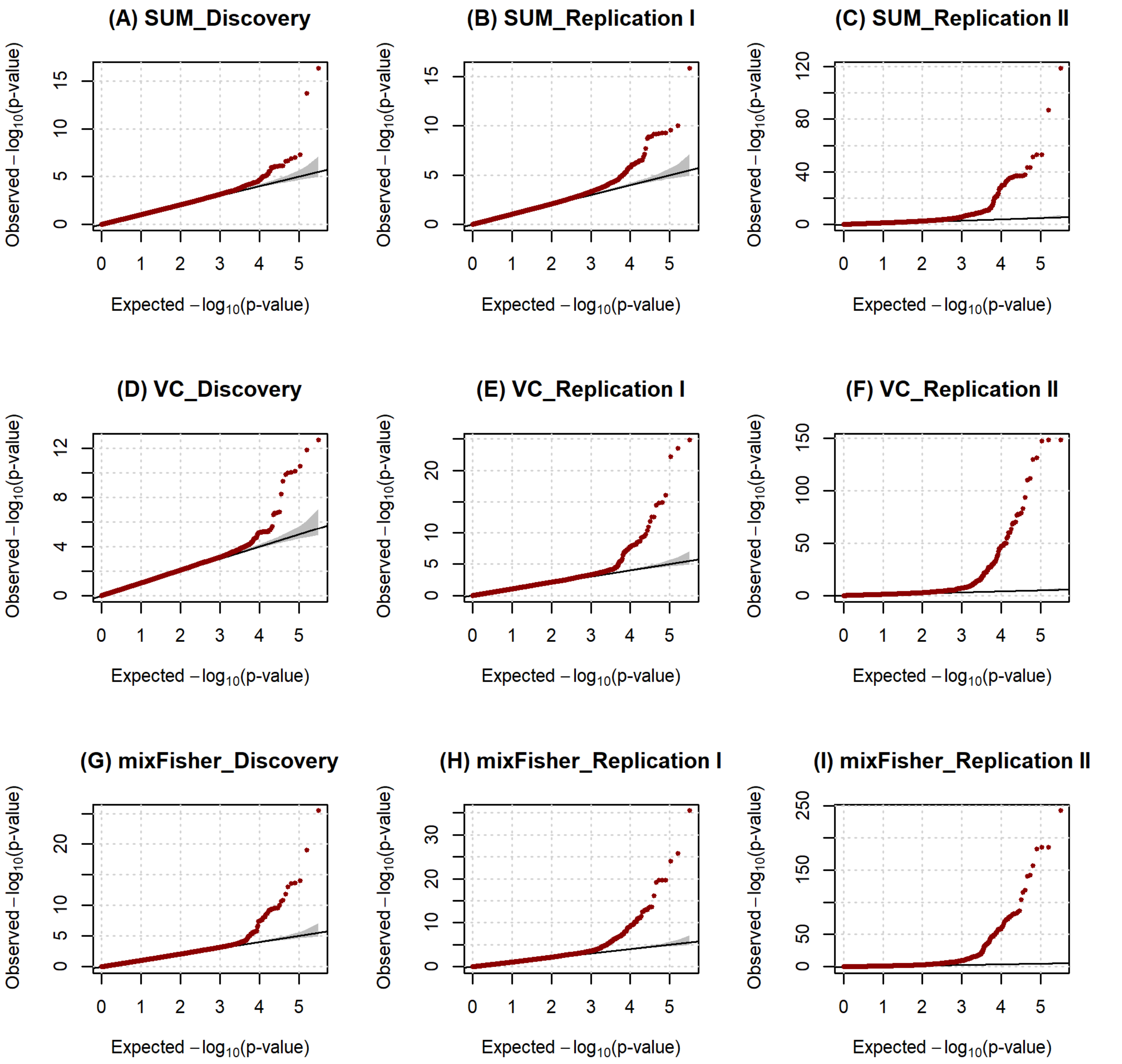

Figure S2. Quantile–quantile plots for the multivariate Multiple Phenotype Association Test analyses in the discovery and two replication cohorts. Panels (A–C), (D–F), and (G–I) correspond to the SUM, VC, and mix-Fisher statistics, respectively, each shown for the discovery, replication I, and replication II cohorts.

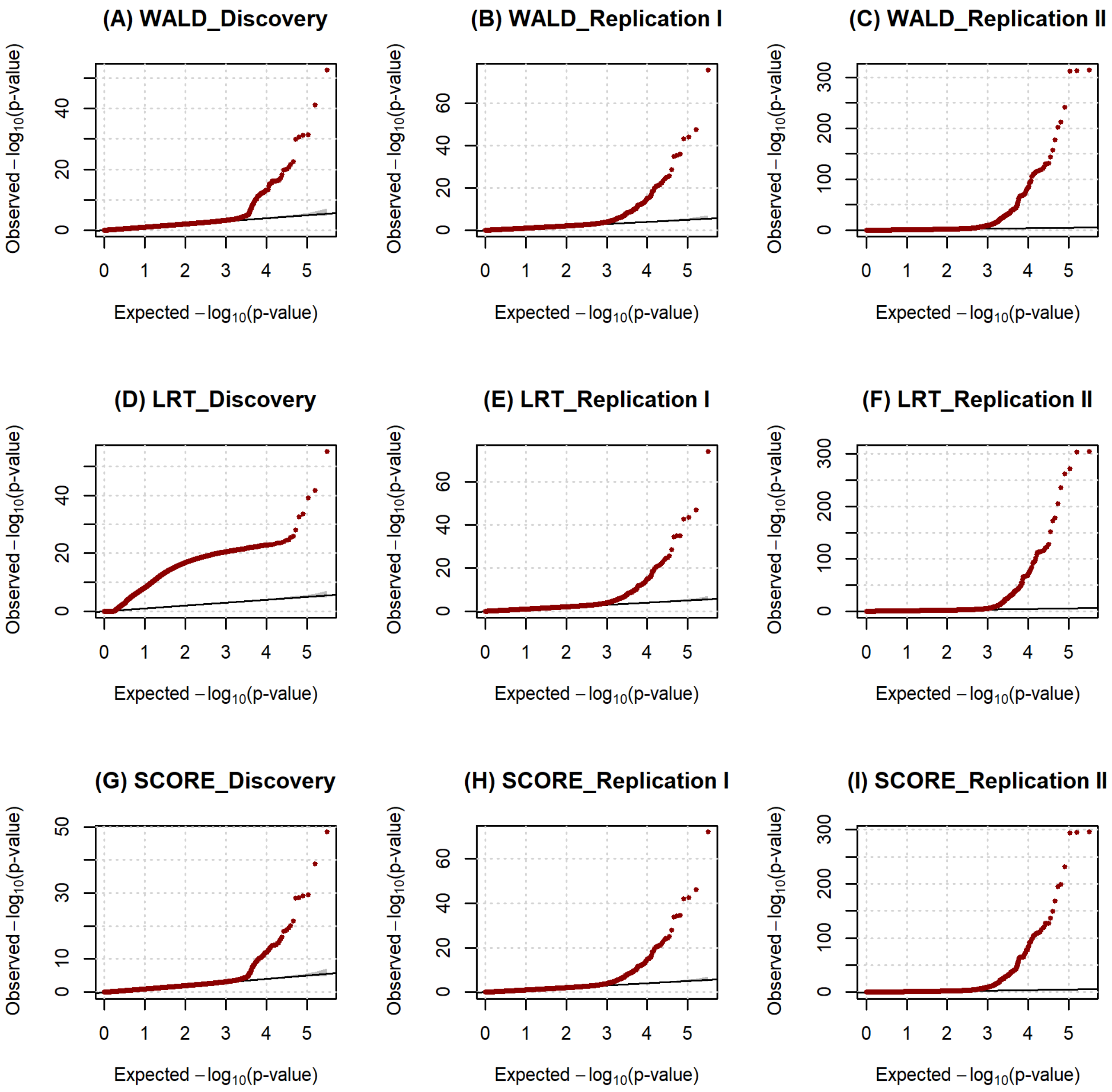

Figure S3. Quantile–quantile plots for the multivariate Genome-wide Efficient Mixed Model Association analyses in the discovery and two replication cohorts. Panels (A–C), (D–F), and (G–I) correspond to the WALD, LRT, and SCORE statistics, respectively, each shown for the discovery, replication I, and replication II cohorts.
